# Whole-exome sequencing identifies two novel mutations in *KCNQ4* in individuals with nonsyndromic hearing loss

**DOI:** 10.1038/s41598-018-34876-9

**Published:** 2018-11-09

**Authors:** Jinsei Jung, Hyun Been Choi, Young Ik Koh, John Hoon Rim, Hye Ji Choi, Sung Huhn Kim, Jae Hyun Lee, Jieun An, Ami Kim, Joon Suk Lee, Sun Young Joo, Seyoung Yu, Jae Young Choi, Tong Mook Kang, Heon Yung Gee

**Affiliations:** 1 Department of Otorhinolaryngology, Brain Korea 21 PLUS Project for Medical Sciences, Yonsei University College of Medicine, Seoul, 03722 Korea; 2 Department of Physiology, Sungkyunkwan University School of Medicine, Suwon, 16419 Korea; 3 Department of Pharmacology, Brain Korea 21 PLUS Project for Medical Sciences, Yonsei University College of Medicine, Seoul, 03722 Korea

## Abstract

Mutations in potassium voltage-gated channel subfamily Q member 4 (*KCNQ4*) are etiologically linked to a type of nonsyndromic hearing loss, deafness nonsyndromic autosomal dominant 2 (DFNA2). We performed whole-exome sequencing for 98 families with hearing loss and found mutations in *KCNQ4* in five families. In this study, we characterized two novel mutations in *KCNQ4*: a missense mutation (c.796G>T; p.Asp266Tyr) and an in-frame deletion mutation (c.259_267del; p.Val87_Asn89del). p.Asp266Tyr located in the channel pore region resulted in early onset and moderate hearing loss, whereas p.Val87_Asn89del located in the N-terminal cytoplasmic region resulted in late onset and high frequency-specific hearing loss. When heterologously expressed in HEK 293 T cells, both mutant proteins did not show defects in protein trafficking to the plasma membrane or in interactions with wild-type (WT) KCNQ4 channels. Patch-clamp analysis demonstrated that both p.Asp266Tyr and p.Val87_Asn89del mutant channels lost conductance and were completely unresponsive to KCNQ activators, such as retigabine, zinc pyrithione, and ML213. Channels assembled from WT-p.Asp266Tyr concatemers, like those from WT-WT concatemers, exhibited conductance and responsiveness to KCNQ activators. However, channels assembled from WT-p.Val87_Asn89del concatemers showed impaired conductance, suggesting that p.Val87_Asn89del caused complete loss-of-function with a strong dominant-negative effect on functional WT channels. Therefore, the main pathological mechanism may be related to loss of K^+^ channel activity, not defects in trafficking.

## Introduction

Hearing loss, a common sensorial disorder with an incidence of 1 in 500–1000 individuals worldwide^1^, is genetically and clinically heterogeneous. At least half the cases are attributable to genetic factors, and more than two-thirds of such cases are classified as nonsyndromic hearing loss (NSHL)^2^. Among NSHL, approximately 20% are transmitted as autosomal dominant traits; more than 60 loci for autosomal dominant NSHL (ADNSHL) have been mapped, and about 30 genes have been identified as causing ADNSHL if mutated (http://hereditaryhearingloss.org/). Mutations in *WFS1, KCNQ4, COCH, and TECTA* are frequently detected in individuals with ADNSHL^3^ and may cause mild to moderate hearing loss.

*KCNQ4* (MIM 600101, Kv7.4) is a voltage-gated potassium channel that plays essential roles in maintaining ion homeostasis and regulating hair cell membrane potential^4^. *KCNQ4* mutations cause deafness nonsyndromic autosomal dominant 2 (DFNA2), which is characterized by progressive sensorineural hearing loss at all frequencies^4,5^. *Kcnq4*^−/−^ mice exhibit degeneration of outer hair cells and progressive hearing loss^6^. Approximately 30 mutations in *KCNQ4* have been reported (http://www.hgmd.cf.ac.uk/ac/index.php and https://www.ncbi.nlm.nih.gov/clinvar/, Table S1). KCNQ4 protein consists of six transmembrane domains and pore region^7^, most mutations are in the pore region^8^.

The pathogenic mechanism of hearing loss associated with *KCNQ4* mutation involves inhibition of the normal function of wild-type (WT) KCNQ4^7^. However, several mutations located in the N-terminal cytoplasmic region are related to haploinsufficiency^9,10^. The genotype-phenotype correlation has not been clearly elucidated. Thus, in this study, we performed whole-exome sequencing (WES) to identify novel mutations in *KCNQ4* among Korean families with ADNSHL.

## Methods

### Patients and diagnosis of sensorineural hearing loss

This study was approved by the institutional review board of the Severance Hospital, Yonsei University Health System (IRB#4-2015-0659). All research was performed in accordance with relevant regulations of the Severance Hospital. After obtaining informed consent, individuals with hearing loss were enrolled in the Yonsei University Hearing Loss (YUHL) cohort, and their clinical and pedigree data were recorded. All patients who were registered in YUHL cohort had bilateral hearing loss and were referred to Severance Hospital for further evaluation and treatment (n = 342). Pure tone audiogram and auditory brainstem response analyses were performed for all patients and their unaffected family members. We obtained audiometric data for all participants. Pure-tone air (250–8000 Hz) and bone conduction (250–4000 Hz) thresholds were measured with clinical audiometers in a double-walled audio booth. The degree of hearing loss was determined by averaging the thresholds at 500, 1000, 2000, and 4000 Hz of air conduction. In addition, temporal bone computed tomography and magnetic resonance imaging were performed.

### DNA preparation, WES, sequence alignment, and variant calling

Whole blood (3 ml) was obtained from the affected individuals and their parents. Genomic DNA was extracted from peripheral leukocytes using RBC Lysis Solution, Cell Lysis Solution, and Protein Precipitation Solution (iNtRon Biotechnology, Inc). Whole-exome capture was performed using an Agilent SureSelect V5 enrichment capture kit (Agilent Technologies, Santa Clara, CA, USA), and the enriched library was then sequenced using an Illumina HiSeq. 2500 instrument (101 bases paired end). Variant filtering was carried out as described previously^11^. In the first step, variants with minor allele frequencies >1% in the gnomAD database (http://gnomad.broadinstitute.org/) were excluded. In the second step, variants present in the homozygous or hemizygous state in 32 healthy Korean individuals without hearing loss (internal control WES data) were excluded. In the third step, synonymous variants and intronic variants not located within the splice site regions were excluded. In the fourth step, variants of all 144 genes known to be monogenic factors for hearing loss were systematically evaluated (Table S2). The process for variant filtering is described in Table S3.

### Three-dimensional structure modeling

To examine structural changes in KCNQ4 protein, three-dimensional protein modeling was performed for the ion transport domain of KCNQ4. A BLAST sequence search against the protein data bank (PDB) was performed to select the template structure with the closest sequence similarity to the domain of KCNQ4. The structural model for the full-length SHAKER potassium channel Kv1.2 from *Rattus norvegicus* (PDB ID: 3LUT) was then selected. The sequence similarity between these domains was 33%. SWISS-MODEL was used to generate the tertiary structure of the domains (SWISS-MODEL, http://swissmodel.expasy.org/). Modeling of the ion transport domain-p.Asp266Tyr was based on the PDB template files. Molecular graphics and analyses were performed using the USCF Chimera package (Chimera, http://www.cgl.ucsf.edu/chimera).

### Plasmid construction and site-directed mutagenesis

cDNAs for human *KCNQ4* were purchased from OriGene Technologies (Rockville, MD, USA). cDNA was subcloned into the pENTR-D-TOPO vector (Invitrogen, Carlsbad, CA, USA). Expression vectors were created using LR clonase (Invitrogen) following the manufacturer’s instructions. Clones reflecting the *KCNQ4* mutations identified in individuals with NSHL were introduced in the cDNA constructs in the pENTR-D-TOPO vector using a Quick change II XL site-directed mutagenesis kit (Agilent Technologies). Tandem concatemers of KCNQ4 WT subunits or of one WT and one mutant subunit were generated by fusing the subunits C-terminus to N-terminus.

### Cell culture and transfection

Human embryonic kidney 293 T (HEK293T) and HeLa cells were cultured in Dulbecco’s modified Eagle medium supplemented with 10% fetal bovine serum and penicillin (50 IU/ml)/streptomycin (50 μg/ml; Invitrogen). The cells were transfected with WT or mutant *KCNQ4* plasmids using Lipofectamine PLUS reagent (Invitrogen) according to the manufacturer’s instructions. To analyze the electrophysiological properties of homotetrameric KCNQ4 channels, KCNQ4 WT, p.Asp266Tyr, and p.Val87_Asn89del were cloned into the pRK5 vector and transiently expressed with pEGFPN-1 in HEK293T cells. Similarly, three tandem concatemers fused with WT KCNQ4 (WT-WT, WT-p.Asp266Tyr, and WT-p.Val87_Asn89del) were cloned into the pRK5 vector and transiently expressed in HEK293T cells with pEGFPN-1. HEK293T cells transfected with empty pRK5 vectors and green fluorescent protein (GFP) were used as a control group.

### Immunoblotting, immunoprecipitation, surface biotinylation, and immunofluorescence

Experiments were performed as described previously^12^. Anti-binding immunoglobulin protein (BiP; ab21685; Abcam, Cambridge, UK), anti-β-actin (ab6276; Abcam), anti-Myc (sc-40; Santa Cruz Biotechnology, Santa Cruz, CA, USA), anti-adolase A1 (sc-12059; Santa Cruz Biotechnology), anti-golgin B1 (GOLGB1; HPA011008, Sigma, St. Louis, Mo, USA), and anti-FLAG (F3165; Sigma) antibodies were purchased from commercial sources. Co-immunoprecipitation was performed using EZview Red Anti-FLAG M2 and Anti-c-Myc Affinity Gel (Sigma). Surface biotinylation was performed using 0.3 mg/ml EZ-Link Sulfo-NHS-SS-Biotin and NeutrAvidin (Thermo Scientific). Immunoblotting was performed using primary antibodies at 1:1000 dilution followed by the corresponding anti-isotype secondary antibodies (Santa Cruz Biotechnology) at 1:2000 dilution. Signals were visualized using the SuperSignal West-Pico kit (Thermo Scientific). Confocal images were obtained with a Carl Zeiss LSM780 microscope, and ZEN was used for image analysis.

### Whole-cell patch clamp assays

KCNQ4 ionic currents were measured with a conventional whole-cell patch clamp technique. HEK293T cells were transiently transfected with *KCNQ4* plasmids using Lipofectamine and seeded onto poly-l-lysine-coated recording chambers mounted on an inverted microscope (IX-70; Olympus, Japan). A healthy-looking cell with green fluorescence (GFP) was selected for whole-cell patch clamp recording. Recording patch pipettes were pulled from borosilicate glass tubing (WPI, Sarasota, FL, USA), and the pipette tip was fire-polished with a microforge (MF-83; Narishige, Japan). The final pipette tip resistance was 1.5–3 MΩ. After achieving conventional whole-cell patch clamp configuration, whole-cell K^+^ currents were amplified and recorded with an Axopatch-1D amplifier (Axon Instrument, USA). The currents were filtered at 5 KHz and acquired at a sampling rate of 10 kHz. Before acquiring ionic currents, series resistance was compensated, and the cell membrane capacitance (C_m_) was measured and cancelled using a circuit of the patch-clamp amplifier. K^+^ currents were generated with 2-s depolarizing voltage steps ranging from −70 to +40 mV in 10-mV increments, followed by a 1-s hyperpolarizing voltage step to −50 mV. Step voltage pulses were generated every 10 s. All recordings were performed at room temperature (~23 °C). Recorded currents were analyzed using Clampex software (pCLAMP 7.0; Axon Instrument). To isolate KCNQ4-mediated K^+^ current from the total whole-cell currents, linopirdine (30 μM) was administrated into the cells, and the linopirdine-sensitive component was obtained by digital subtraction. For comparison, KCNQ4 current densities (pA/pF) were calculated by dividing the KCNQ4 steady-state current amplitudes recorded at +40 mV with the measured C_m_. The relationship between normalized conductance (G/G_max_) and voltage (V) was calculated from the I-V curve of the current, and the half-activation voltage (V_1/2_) of the channel was calculated by fitting to a Boltzmann function.

### Solutions and chemicals used for patch clamp study

The external bath solution for whole-cell patch clamp recording consisted of 147 mM NaCl, 5 mM KCl, 1.5 mM CaCl_2_, 1 mM MgCl_2_, 10 mM HEPES, and 10 mM d-glucose, adjusted to pH 7.4 with N-methyl-d-glucamine. The internal patch pipette solution contained 130 mM KCl, 10 mM NaCl, 10 mM EGTA, 10 mM HEPES, 3 mM Mg-ATP, 0.5 mM CaCl_2_, adjusted to pH 7.2 with KOH. The calculated free Ca^2+^ concentration was about 10 nM.

Linopirdine dihydrochloride (Tocris Bioscience, Bristol, UK), retigabine (Glentham Life Science, Corsham, UK), and zinc pyrithione (Sigma-Aldrich, St. Louis, MO, USA), and ML213 (Tocris Bioscience) were dissolved in DMSO and prepared as 5–30 mM stock solutions. Each stock solution was diluted into the external bath solution before use.

### Statistics

The obtained values were expressed as means ± standard errors of the means. The data were plotted and analyzed with Origin software (version 6.1; OriginLab, USA). Statistical significance was determined by paired or unpaired Student’s *t*-tests, and differences with P values of less than 0.05 were considered significant.

## Results

### WES identified autosomal dominant mutations in *KCNQ4*

**Figure 1:**
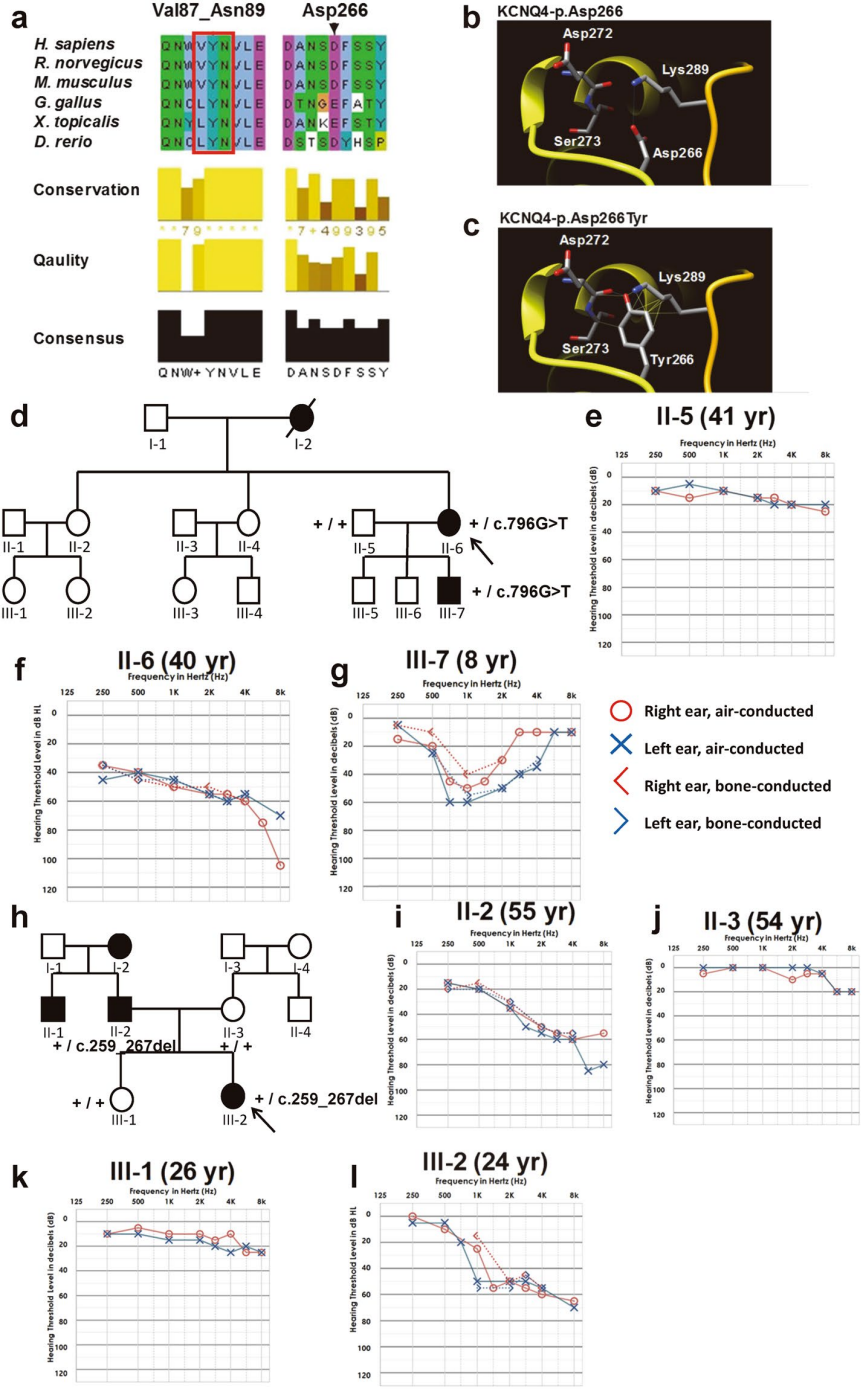
*KCNQ4* mutations identified by whole-exome sequencing, and pedigrees and audiological phenotypes of YUHL35 and YUHL41 families. (**a**) Evolutionary conservation of altered amino acid residues of KCNQ4. (**b,c**) Computational tertiary structure prediction of KCNQ4 wild-type and p.Asp266Tyr proteins. Asp266 shared a hydrogen bond with Lys289 (K), whereas p.Asp266Tyr had hydrogen bonds with Asp272, Ser273, and Lys289 (L). Moreover, Lys289 clashed with the p.Asp266Tyr mutation. YUHL, Yonsei University hearing loss. (**d**) The family pedigree of YUHL35 with autosomal dominant hearing loss. *KCNQ4* genotypes of individuals in whom Sanger sequencing was performed are indicated. (**e–g**) The pure-tone audiogram showed moderate sensorineural hearing loss bilaterally in II-6 (f). Note that the 8-year-old male only had mid-frequency hearing loss up to 60 dB HL (g). (**h**) An autosomal dominant inheritance pattern was observed in the YUHL41 pedigree. *KCNQ4* genotypes of individuals in whom Sanger sequencing was performed are indicated. (**i–l**) Affected individuals II-2 (i) and III-2 (l) had residual hearing function at 250 and 500 Hz, whereas hearing loss at high frequencies was observed in pure-tone audiograms.

342 families with hearing loss have been enrolled in the YUHL cohort, and *GJB2* and *SLC26A4* were examined by Sanger sequencing as these are most common cause of hearing loss in Korea^11^. We performed WES for 98 families in which *GJB2* and *SLC26A4* were excluded. In 38 of 98 families including YUHL35 and YUHL41 (Fig. 1), autosomal dominant inheritance was suspected based on pedigrees. WES detected potentially causative mutations in *KCNQ4* in five families, therefore, 13.1% (5/38) of families suspecting an autosomal dominant inheritance were explained by *KCNQ4* mutations. Missense variant (c.796 G>T; p.Asp266Ty) and an in-frame deletion variant (c.259_267delGTCTACACC; p.Val87_Asn89del) were detected in YUHL35 II-6 and YUHL41 III-2, respectively (Table 1). Other variants in known genes were excluded (Table S4). Both variants in *KCNQ4* have not been reported in any public databases and were segregated with the affected status (Table 1 and Fig. S2). The amino acid residues deleted by the in-frame deletion mutation were conserved throughout evolution (Fig. 1a). Both *KCNQ4* variants identified in this study had not been previously reported, therefore, their pathogenicity should be examined. The other three families shared a same *KCNQ4* mutation which has not been reported yet and we are currently investigating the pathogenicity of this mutation.

**Table 1:** Mutations in *KCNQ4* detected in individuals with nonsyndromic hearing loss by WES.

Gene symbol	Family	Sex	Age of onset	Nucleotide change^a^	Amino acid change	Exon (zygosity, segregation)	GERP^b^	PhyloP^c^	Frequencies in the dbSNP database^d^	Frequencies in the gnomAD database^e^	Frequencies in the NBK database^f^	PP2^g^	MT^h^	PROVEAN^i^	SIFT^j^
*KCNQ4*	YUHL 35	Fm	30 yr	c.796G>T	p.Asp266Tyr	5 (het, ND)	5.08	4.749	ND	ND	ND	PD (0.980)	DC (0.999)	Del (−2.96)	Dam (0.001)
	YUHL 41	Fm	Early 1^st^ decade	c.259_267del GTCTACAAC	p.Val87_Asn89del	1 (het, F)	NA	NA	ND	ND	ND	NA	PM (0.866)	Del (−17.62)	NA

Abbreviations are as follows: Dam, damaging; DC, disease causing; Del, deleterious; F, heterozygous mutation identified in the father; Fm, female; het, heterozygous in the affected individual; M, heterozygous mutation identified in the mother; MT, Mutation Taster; NA, not applicable; ND, no data or DNA available; PD, probably damaging; PM, polymorphism; PP2, PolyPhen-2 prediction score Humvar; PROVEAN, Protein Variation Effect Analyzer; SIFT, Sorting Intolerant from Tolerant; SNP, single nucleotide polymorphism; yr, years; YUHL, Yonsei University Hearing Loss cohort.

^**a**^cDNA mutations are numbered according to the human cDNA reference sequence NM_004700.3 (*KCNQ4*); +1 corresponds to the A of ATG translation initiation codon. ^b^Genomic Evolutionary Rate Profiling (GERP) score. ^b^PhyloP100way_vertebrate score. ^d^dbSNP database (http://www.ncbi.nlm.nih.gov/SNP). ^e^genome Aggregation Database browser (http://gnomad.broadinstitute.org/). ^f^National Biobank of Korea (NBK), Centers for Disease Control and Prevention. ^g^PolyPhen-2 (PP2) prediction score HumVar ranges from 0 to 1.0; 0 = benign, 1.0 = probably damaging (http://genetics.bwh.harvard.edu/pph2/). ^h^Mutation taster (http://www.mutationtaster.org/). ^i^PROVEAN, (http://provean.jcvi.org/index.php). ^j^SIFT, (http://sift.jcvi.org/).

We first performed a computational tertiary structure prediction of WT and mutant KCNQ4 proteins. The Asp residue at position 266 of KCNQ4 protein was in the ion permeating pore domain and formed a hydrogen bond with Lys289 (Fig. 1b). However, the p.Asp266Tyr mutant formed hydrogen bonds with Asp272, Ser273, and Lys289 (Fig. 1c). Moreover, Lys289 clashed with the amino acid substitution from Asp to Tyr at position 266.

### Audiological phenotype and clinical assessment

The pedigree of YUHL35 revealed the autosomal dominant nature of hearing loss (Fig. 1d). Affected patients (II-6 and III-7) had moderate bilateral sensorineural hearing loss; II-6 had down-sloping hearing loss with an acoustic threshold of 50 dB HL through all frequencies, whereas III-7 had only mid-frequency hearing loss up to 60 dB HL (Fig. 1e–g). The ages of onset were the early second and first decades for II-6 and III-7, respectively. II-6 had bilateral progressive hearing deterioration during the follow-up period (Fig. S1). Both II-6 and III-7 had no vestibular symptoms.

In the YUHL41 family, ADNSHL was associated with high-frequency hearing loss (Fig. 1h). Affected individuals had symmetric down sloping hearing loss up to 60 dB HL; hearing functions at 250 and 500 Hz were well preserved in all unaffected cases (Fig. 1i–l). Interestingly, II-2 and III-2 exhibited similar hearing thresholds although there was a three-decade age difference, implying that hearing loss was not progressive.

### Effects of the identified mutations on the surface expression and subunit interactions of KCNQ4

**Figure 2:**
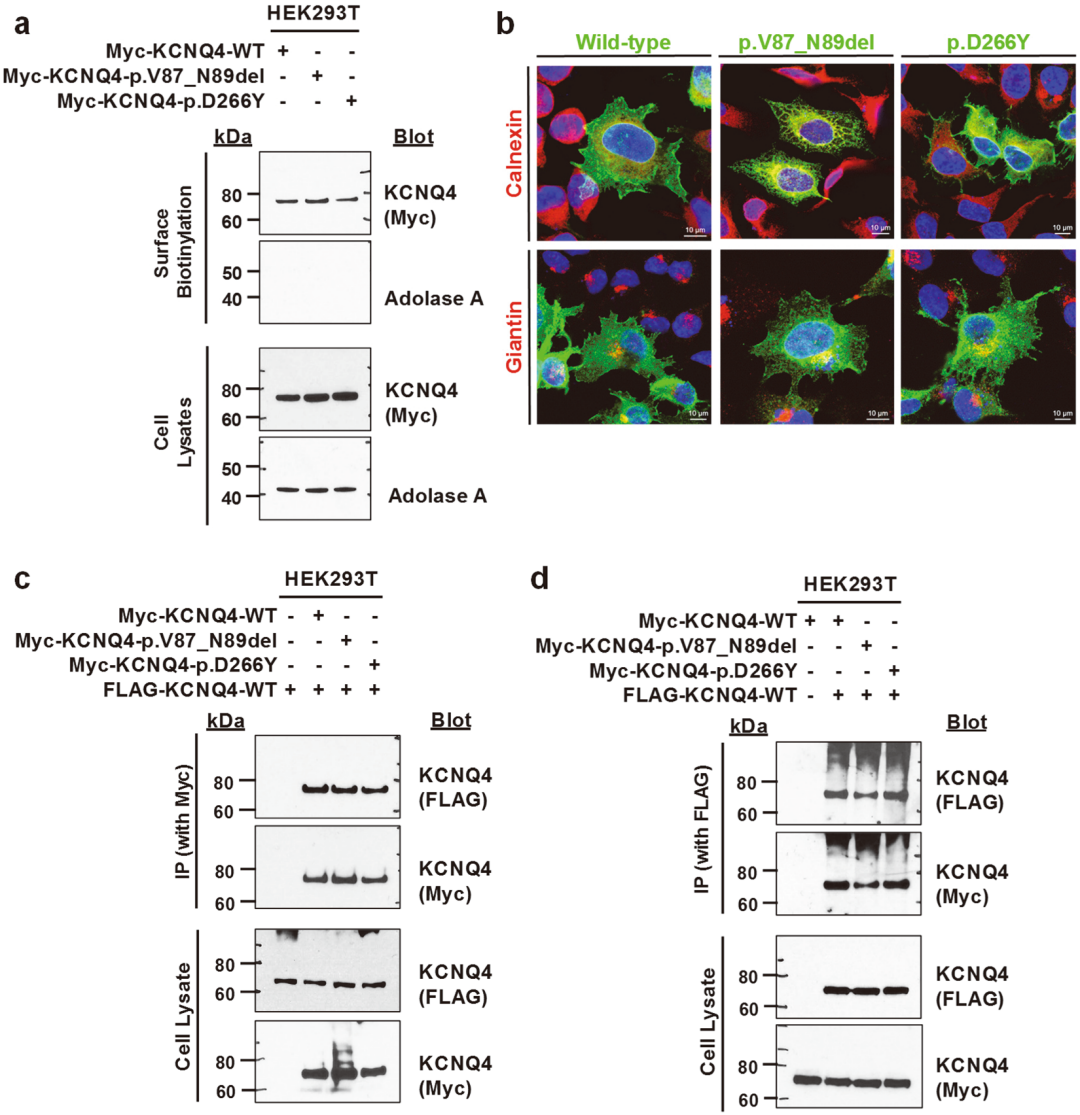
Surface expression and subunit interactions of WT and mutant KCNQ4 proteins. HEK293T or HeLa cells were transfected with N-terminally Myc- or Flag-tagged WT and mutant KCNQ4 clones. (**a**) Cell surface biotinylation in HEK293T. Proteins on the plasma membrane were labelled with biotin, isolated with avidin beads, and assessed by western blotting. Surface expression of two mutant KCNQ4 proteins was similar to that of WT protein. (**b**) Immunofluorescence of WT and mutant KCNQ4 proteins in HeLa cells. Cells were immunostained with anti-Myc, anti-calnexin, and anti-giantin antibodies. Nuclei were stained with DAPI. Calnexin and giantin are markers for the endoplasmic reticulum and Golgi apparatus, respectively. Both mutant KCNQ4 proteins and WT protein were observed on the plasma membrane. (**c–d**) Subunit interactions between WT and mutant KCNQ4 proteins. Twenty-four hours post-transfection, whole-cell lysates were subjected to immunoprecipitation using anti-Myc (**c**) or anti-FLAG (**d**) antibodies and immunoblotted. Both KCNQ4 mutant proteins interacted with WT protein.

To evaluate the impact of the identified mutations on trafficking of KCNQ4 to the plasma membrane, we performed surface biotinylation of WT and mutant KCNQ4 proteins in HEK293 cells. When overexpressed, both mutant proteins and the WT protein showed cell surface expression (Fig. 2a). The absence of the cytosolic protein aldolase A in the biotinylated fraction confirmed cell surface protein-specific labeling. In addition, there were no differences in expression levels between WT and mutant proteins.

Immunofluorescence analysis showed that both mutant proteins and the WT protein were localized to the plasma membrane (Figs 2b and S3). p.Val87_Asn89del mutant protein reached the cell surface, although a significant portion of the protein seemed to be trapped in the endoplasmic reticulum (ER) or Golgi apparatus, as shown by colocalization with the ER marker Calnexin and Golgi marker Giantin, respectively (Fig. 2b). The Val-Tyr-Asn residues at positions 87–89 of KCNQ4 are also conserved in KCNQ1 and these amino acid residues are a part of the N-terminal juxtamembranous domain which is critical for surface expression of KCNQ1^13^. Therefore, we compared p.Val87_Asn89 mutant protein with a known trafficking mutant (ΔF508) of cystic fibrosis transmembrane conductance regulator (CFTR)^14^. The expression level of ΔF508-CFTR was significantly increased by the proteosomal inhibitor MG132, whereas those of WT-CFTR, WT and mutant KCNQ4 proteins were not affected by MG132 (Fig. S4). The amount of p.Val_Asn89del mutant protein was not changed by the lysosomal inhibitor leupeptin (Fig. S4a).

The functional potassium channel is formed by a tetrameric assembly of KCNQ4 subunits^9^. Individuals with *KCNQ4* mutations were heterozygous (Fig. S2), and expression levels of mutant proteins were comparable to that of the WT protein (Fig. 2a). Therefore, WT or mutant Myc-tagged KCNQ4 clones were cotransfected with the WT FLAG-KCNQ4 clone into HEK293T cells. The heteromeric assembly was analyzed by co-immunoprecipitation. Similar to WT protein, both p.Val87_Asn89del and p.Asp266Tyr proteins showed normal ability to interact with WT protein, suggesting that tetramerization between WT and mutant proteins was unlikely to be disrupted (Fig. 2c,d).

### Effects of the identified mutations on KCNQ4 channel conductance

**Figure 3:**
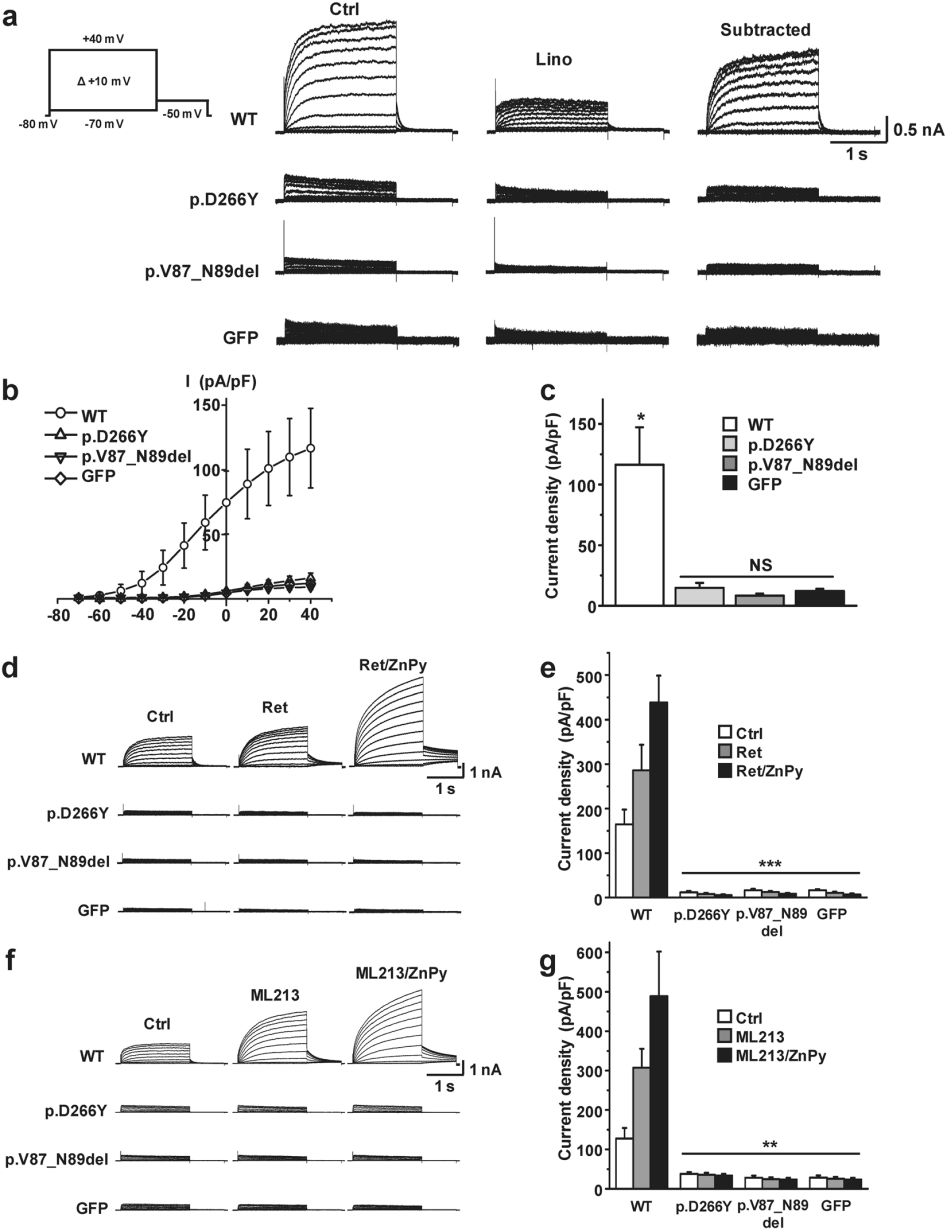
Impaired potassium conductance of homomeric mutant KCNQ4 channels. (**a**) Whole-cell KCNQ4 K^+^ current traces recorded from the HEK293T cells transiently expressing WT, p.D266Y, p.V87_N89del, or GFP. Homomeric p.Asp266Tyr and p.Val87_Asn89del mutant channels produced barely detectable K^+^ currents. (**b,c**) Current-voltage (I-V) relationships of linopirdine (30 μM)-sensitive K^+^ currents (**b**), and current densities measured at +40 mV (c). The I-V curve of the WT protein exhibited a typical outwardly rectifying KCNQ4 channel current, and the current densities of p.D266Y and p.V87_N89del mutants were not significant (NS) compared with GFP. WT, n = 24; p.D266Y, n = 10; p.V87_N89del, n = 10; GFP, n = 22. *P < 0.05 versus mutants and GFP. (**d–g**) Homomeric mutant channels were not activated by known KCNQ openers. Single or combination treatment with retigabine (Ret, 10 μM), ML213 (3 μM), and zinc pyrithione (ZnPy, 10 μM) did not activate mutant channels (**d,f**), and the current densities after treating KCNQ openers were not significantly different from that of GFP (**e,g**). Mean ± SEM, n = 6–9; **P < 0.01 and ***P < 0.005 versus the WT protein.

Ion conductance of homomeric mutant channels was compared with that of the functional WT KCNQ4 channel. In conventional whole-cell patch clamp recordings, KCNQ4 mutant channels could not generate appreciable K^+^ currents (Fig. 3). After subtraction of the linopirdine-sensitive component from the total K^+^ current, the I-V curve of the WT KCNQ4-mediated K^+^ current showed a typical curve of KCNQ4 channels (Fig. 3a,b). However, similar to GFP-transfected cells, p.Asp266Tyr and p.Val87_Asn89del mutant channels produced barely detectable currents (Fig. 3c). Furthermore, these two mutant channels were not activated by known KCNQ openers (10 μM retigabine, 3 μM ML213, and 10 μM zinc pyrithione)^15–18^. Combination of retigabine or ML213 with zinc pyrithione further increased WT-mediated current in an additive manner. However, none of the combinations activated the current in either mutant (Fig. 3d–g). These findings suggest that both p.Asp266Tyr and p.Val87_Asn89del channels were loss-of-function mutants.

### Dominant-negative effects of the mutant KCNQ4 channels

**Figure 4:**
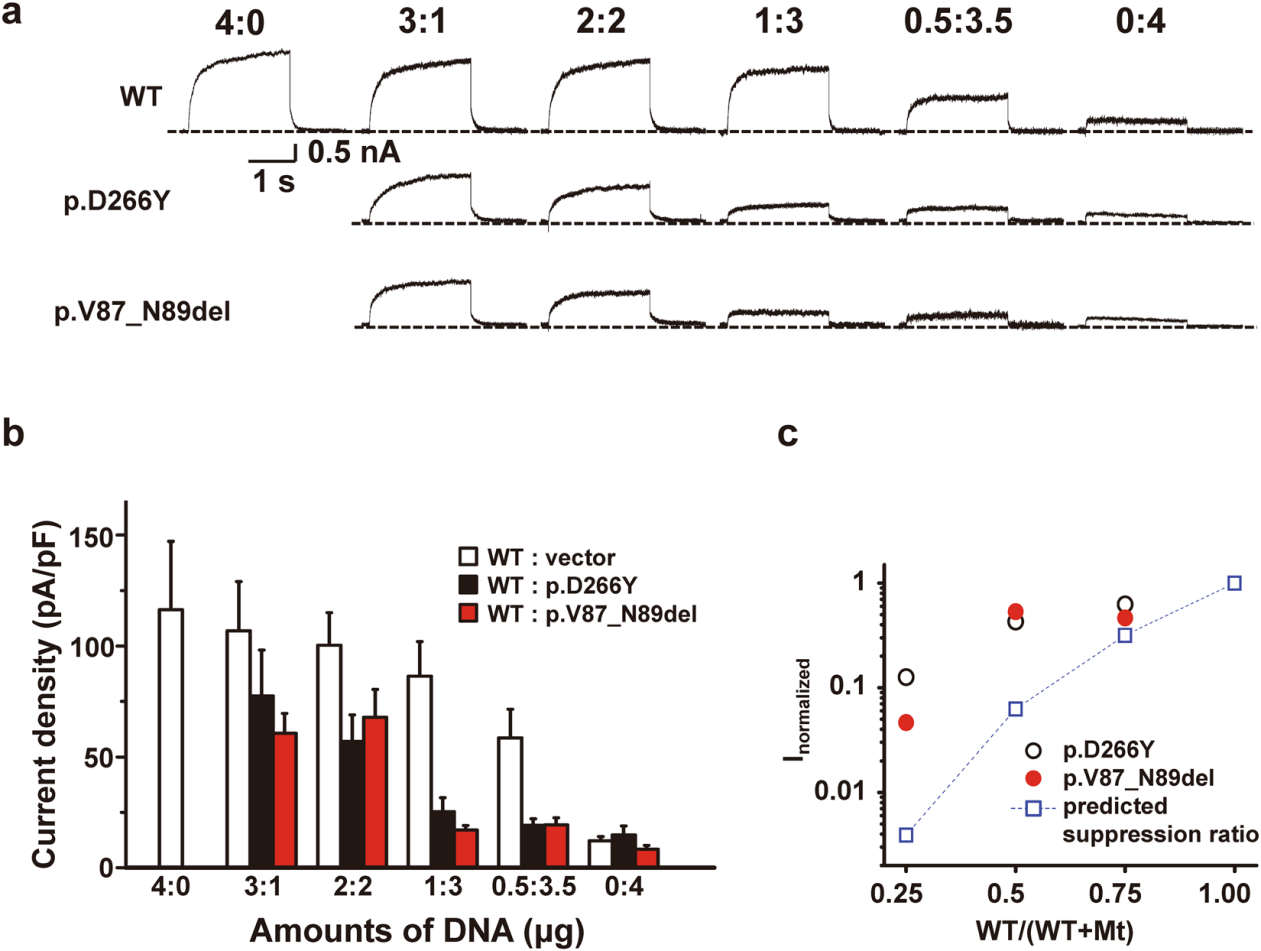
Dominant-negative effects of the mutant KCNQ4 channels. (**a**) Individual mutants (p.D266Y and p.V87_N89del) were co-expressed with WT KCNQ4 at the indicated WT:mutant cDNA molar ratios, and linopirdine-sensitive K^+^ current traces were recorded at +40 mV. Dashed lines indicate zero current levels. (**b**) Comparison of current densities at +40 mV. WT:mutant cDNA ratios are indicated under the bar graphs, and the total amount of cDNA was equalized in all groups by adding empty vector (pRK5). (**c**) Suppression of WT-mediated current by the co-expression of mutant (Mt) KCNQ4 channels. The mean values of the current densities obtained at +40 mV were normalized, and the current suppression ratios were denoted against WT/(WT + mutant) ratios of cDNA transfected. Dashed line with a square symbol denotes the predicted suppression ratio expected for the tetrameric channel. Mean ± SEMs (n = 10–24).

To evaluate the dominant-negative effects of the mutant channels, p.Asp266Tyr and p.Val87_Asn89del mutants were individually co-expressed with the WT KCNQ4 channel at different cDNA molar ratios (WT:mutants ratios ranged from 4:0 to 0:4). By increasing the amounts of mutant cDNA (p.Asp266Tyr or p.Val87_Asn89del), recorded linopirdine-sensitive K^+^ currents were gradually reduced. Below WT:mutants cDNA ratios of 1:3, the measured current size was not significantly different from that of cells with the empty vector (Fig. 4a,b). The measured KCNQ4-mediated K^+^ currents at different WT:mutant ratios were deviated from the predicted suppression ratios for a tetrameric co-assembly of WT with dominant-negative KCNQ4 subunits (Fig. 4c). This result did not support the notion that individual mutant channels had classical dominant-negative effects. However, the variance and outliers in current density values within the same group made it difficult to reach a definitive conclusion.

**Figure 5:**
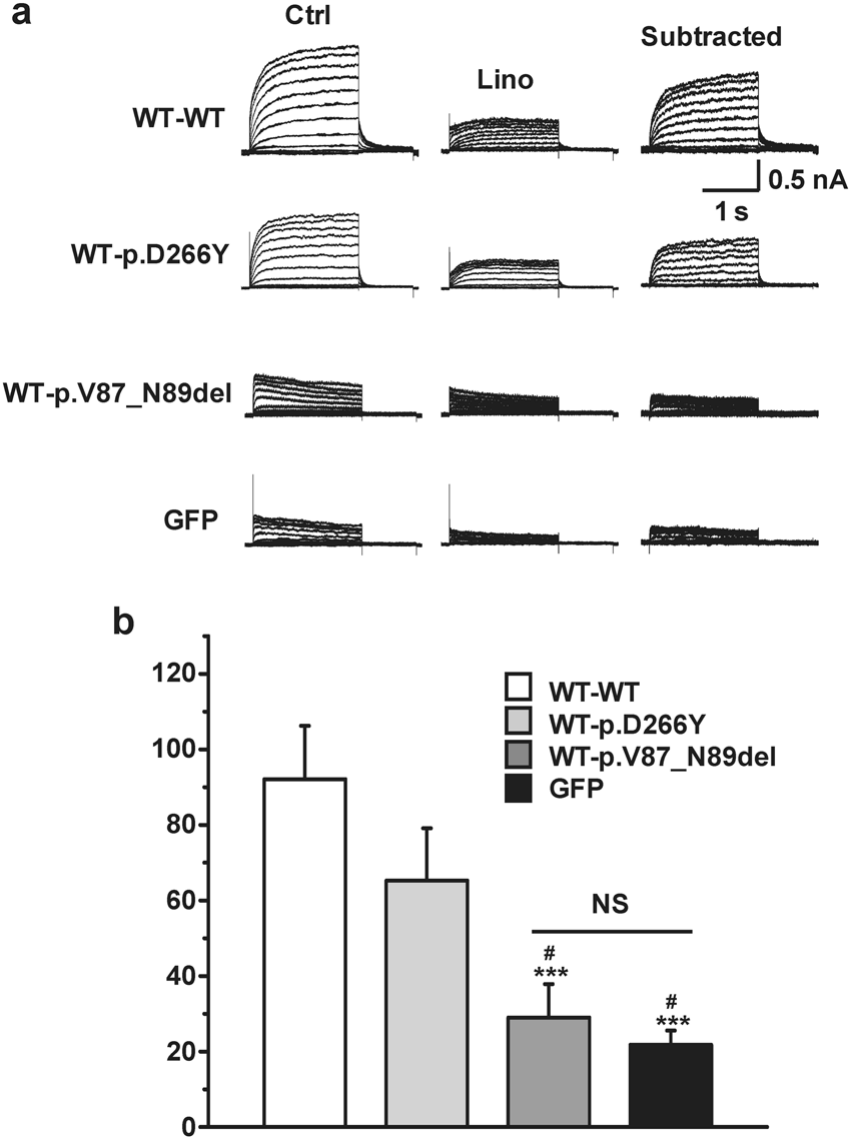
Potassium conductance of KCNQ4 channels assembled from tandem concatemers. (**a**) Representative K^+^ current traces recorded from the KCNQ4 channels assembled from WT-WT, WT-p.D266Y, and WT-p.V87_N89del concatemers. GFP-transfected cells were used as controls, and linopirdine-sensitive currents were subtracted for comparison. (**b**) Comparison of current densities at +40 mV. Means ± SEMs; WT-WT, n = 13; WT-p.D266Y, n = 12; WT-p.V87_N89del, n = 11; GFP, n = 6. NS, not significant; ***p < 0.005 versus WT-WT; #p < 0.05 versus WT-D266Y.

To overcome this limitation, we generated tandem concatemers of the mutants fused with the C-terminal end of the WT channel and examined whether these mutant channels had dominant-negative effects with a forced 2:2 assembly. Importantly, protein synthesis and surface expression of the concatemers were not defective (Fig. S5). In patch-clamp assays, the linopirdine-sensitive K^+^ current generated by the control WT-WT concatemer was nearly equivalent to the current size of homomeric WT channels (Fig. 5). Although homomultimeric p.Asp266Tyr channels had impaired conductance (Fig. 3a), KCNQ4 channels assembled from the WT-p.Asp266Tyr concatemer exhibited 70% of the linopirdine-sensitive K^+^ current of channels assembled from WT-WT concatemers (Fig. 5b). Thus, forced 2:2 assembly of WT and p.Asp266Tyr mutant subunits almost completely restored channel conductance. In contrast, channels assembled from WT-p.Val87_Asn89del concatemers exhibited impaired conductance similar to p.Val87_Asn89del homomultimeric channels and produced a negligible current size similar to that of GFP-transfected cells (Figs 3 and 5). Therefore, we concluded that the p.Val87_Asn89del mutant had strong dominant-negative effects on functional WT channels.

Pharmacological responses to KCNQ activators (retigabine, ML213, zinc pyrithione) were similar between WT-p.Asp266Tyr and WT-WT concatemers (Fig. S6). Single or combination treatments of KCNQ activators increased the WT-WT and WT-p.Asp266Tyr concatemer-mediated current to a similar extent (Fig. S6b). Every KCNQ opener used in this study shifted the activation curves of the WT-WT and WT-p.Asp266Tyr concatemer channels to the hyperpolarizing voltage. The extent of hyperpolarizing movement of half-activation voltages (V_1/2_) was similar between these two concatemers (Fig. S6c,d). In contrast, WT-p.Val87_Asn89del concatemers were not activated by any combination of KCNQ4 openers (Fig. S6a,b). Consistent with this finding, no shift or movement in the activation curves of the WT-p.Val87_Asn89del was detected.

Taken together, our patch-clamp study suggested that the new mutations p.Asp266Tyr and p.Val87_Asn89del in *KCNQ4* resulted in complete loss-of-function and that p.Val87_Asn89del exerted dominant-negative effects on WT channels.

## Discussion

We identified novel mutations (c.796G>T; p.Asp266Ty and c.259_267delGTCTACACC; p.Val87_Asn89del) in *KCNQ4* in patients with NSHL. The position of the affected amino acid residues was well conserved across species.

DFNA2-associated hearing loss involves pathogenic mutations in *KCNQ4*^7,19^. To date, there are 28 pathogenic *KCNQ4* mutations associated with DFNA2 (http://deafnessvariationdatabase.org/). Most mutations are found in a single family, and some recurrent mutations, such as p.Trp276Ser, have been designated by the term “hot spot mutations” in *KCNQ4*^4,7,9,20–23^. However, in a recent report, c.211delC was identified as a recurrent mutation due to the common ancestor effect in Japanese individuals^21^. Indeed, c.211delC mutations were found in seven of 13 families with DFNA2-associated *KCNQ4* mutations. Notably, the prevalence of mutations in *KCNQ4* is as high as 6.67% in Japan and in our YUHL cohort, KCNQ4 mutations were detected in families in which autosomal dominant inheritance was suspected. Thus, screening for mutations in *KCNQ4* should be considered to identify causative mutations in ADNSHL.

The main pathogenic mechanism of mutations in *KCNQ4* is the dominant-negative effect of the mutant protein^6,7^. Because KCNQ4 potassium channels form homotetramers^6,9^, 1/16 [(1/2)^4^] of potassium channels can function normally, possibly contributing to slow progressive hearing loss in patients with heterozygous mutations in *KCNQ4*. In knock-in mouse models and heterologously expressed cell line models, missense mutations were found to inhibit KCNQ4 channel function through dominant-negative effects^6,24^. Specifically, missense mutations in the pore region of KCNQ4 result in early-onset disease and severe hearing loss^9,25^. However, some deletion mutations are correlated with late onset and mild hearing loss, implying that haploinsufficiency is the pathogenic mechanism of DFNA2^9,10^. Thus, the mild phenotype of DFNA2 is likely correlated with truncating mutations in the N-terminal cytoplasmic domain due to the null function of mutant proteins without interfering with WT KCNQ4.

In this study, we found an in-frame three-amino acid deletion mutation (p.Val87_Asn89del) in the N-terminal cytoplasmic domain of KCNQ4, which caused high frequency-specific hearing loss with normal hearing at low frequency (YUHL41 family). Additionally, p.Val87_Asn89del mutant KCNQ4 had a strong dominant-negative effect in electrophysiological studies, which likely caused high frequency-specific hearing loss. Given that KCNQ4 in outer hair cells is evenly expressed in both basal and apex turns of the cochlea, high frequency-specific hearing loss is hardly explained only by changes in the expression of KCNQ4 in the outer hair cells. In addition, some patients with DFNA2 show both low and high frequency-hearing loss; therefore, the genotype-phenotype correlation in patients with DFNA2 is highly complex. This complex nature of the phenotypes in DFNA2 may be attributable to the gradient expression of KCNQ4 in spiral ganglion cells as well as in inner hair cells^26,27^.

As in many voltage-gated ion channels, the highly conserved cytoplasmic N-terminal S0 segment is found in KCNQ family channels. The S0 helix segment is considered as a critical scaffolding element that contributes to the structural stability and dynamics of other voltage sensor domains (S1–S4 segments)^28,29^. A number of cardiac long QT syndrome (LQTS)-associated *KCNQ1* variants located in the S0 segment dysregulate channel function by impairing surface expression or by negatively affecting channel conduction. In particular, *KCNQ1* mutations in the C-terminal half of the S0 segment tend to produce severe defects of protein expression or trafficking^29^. In our study, the three deleted amino acids (Val87-Tyr88-Asn89) of the p.Val87_Asn89del mutation are also located in the middle of the S0 segment, and these conserved amino acids correspond to Val110-Tyr111-Asn112 of the KCNQ1 S0 segment^13,29,30^. Therefore, we suspected a trafficking defect in the p.Val87_Asn89del protein. However, no defect in the surface expression of p.Val87_Asn89del protein or the formation of heteromultimeric channels with WT channels were observed. Consistent with these studies, the WT-p.Val87_Asn89del concatemer channels were well detected on the cell surface (Fig. S5).

Instead of defect in membrane expression, electrophysiological studies demonstrated that p.Val87_Asn89del is a loss-of-function mutant and its strong dominant-negative effect impairs the channel conductance of heteromeric channels. Deleted amino acids in the S0 segment may disrupt the stability of the voltage sensor domain of the channel, thereby impairing the voltage-sensitive gating mechanism. The ineffectiveness of KCNQ openers in p.Val87_Asn89del channels may indicate disruption of channel gating properties because the same openers shifted the gating voltages of functional channels to the hyperpolarized voltage range (Fig. S6). Cryo-EM structure and molecular dynamics simulation of the KCNQ1 channel revealed a strong motional coupling between the scaffolding S0 segment, the C-terminal half of S2, and the critical voltage-sensing S4 segment^28,29^. These studies emphasized that the S0 segment contributes to the organization and stabilization of the structure of other voltage sensor domains (S1-S4). We speculate that the p.Val87_Asn89del mutation causes unavoidable structural disruption of the S0 segment and destabilizes the interaction with the voltage sensor, thereby failing to facilitate voltage sensing and gating. The observed dominant negative effect of the p.Val87_Asn89del mutant can be explained by the same principle, i.e., co-assembly of the broken voltage sensor subunit impairs the whole gating process of the tetrameric channel. Cryo-EM structure of KCNQ1 unveiled a unique hinge structure located between S4 and S4–S5 linker^28^. In the absence of membrane lipid phosphatidylinositol 4,5-bisphosphate (PIP_2_) signaling, the hinge permits uncoupling of activated voltage sensors and the closed pore. Therefore, the hinge is an important structure for the regulation of PIP_2_-dependent KCNQ channel gating. From the molecular dynamics study of KCNQ1, two residues in the hinge were also found to be S0 segment-interacting sites^29^. The c.664_681del (p.G215_L220del) KCNQ4 mutant was reported previously as a pathogenic mutation in Korean DFNA patients^24^. Like the p.Val87_Asn89del mutant, this dominant-negative mutation resulted in a loss-of-function channel, but it showed normal cell surface expression. The p.G215_L220del mutation is located in the hinge that interacts with the voltage-sensing S0 segment. Given that these two mutations showed identical biochemical and electrophysiological features (normal protein synthesis, assembly and trafficking of the impaired voltage sensing channels), we suggest that they are good examples to emphasize the pathogenic effect of the impaired voltage sensor of KCNQ4 channel from a perspective of the interaction between the S0 segment and the hinge.

KCNQ4 mutated at the channel pore region produces severely impaired nonconducting channels causing severe-to-profound hearing loss in patients with DFNA2. Missense mutations at the K^+^ ion selectivity filter with the GYG signature sequence (p.Gly285Cys, p.Gly285Ser) or pore-helix domain (p.Leu274His, p.Trp276Ser) showed complete loss of ion conduction and decreased surface expression. Thus, these mutants exhibit strong dominant-negative effects to WT protein, and their impaired conductance was not rescued by co-assembly with conducting WT subunits^4,5,18,21,31^. In the YUHL35 family, the novel KCNQ4 missense mutation at the K^+^ channel pore forming region (p.Asp266Tyr) produced non-conducting homotetrameric KCNQ4 channels and decreased the sensitivity to KCNQ channel openers, but did not affect membrane trafficking or the formation of heteromultimeric channels with WT subunits. In addition, the channels assembled from WT-p.Asp266Tyr concatemers showed conductance comparable to channels assembled from WT-WT concatemers. Because p.Asp266Tyr is located at the outer position of the pore loop with preserved normal sequences at the core pore domain, it can be speculated that this mutant has the capability to recover its ion conduction unlike other pore mutations, by forming heterotetrameric channels with the WT protein. This may explain the relatively mild hearing loss in patients with the p.Asp266Tyr mutation, whereas individuals with missense mutations in the core pore domain, such as p.Trp276Ser, manifest severe-to-profound hearing loss^4,7,9,20–23^. It needs further confirmation whether the p.Asp266Tyr mutation is truly associated with hearing loss.

In conclusion, WES of patients with ADNSHL identified two novel KCNQ4 mutations (p.Asp266Tyr and p.Val87_Asn89del) associated with different audiological phenotypes of DFNA2. Electrophysiology studies demonstrated the pathogenicity of these KCNQ4 mutations, indicating causality for ADNSHL.

## Electronic supplementary material

Supplementary information
