# Supplementary material for: Whole-exome sequencing identifies two novel mutations in KCNQ4 in individuals with nonsyndromic hearing loss
Source: Sci Rep. 2018 Nov 9;8:16659. doi: 10.1038/s41598-018-34876-9 (PMC6226507; doi:10.1038/s41598-018-34876-9)
Supplement: Supplementary file 1 — Supplementary information [file 41598_2018_34876_MOESM1_ESM.pdf]

Supplementary Information

Whole-exome sequencing identifies two novel mutations in *KCNQ4* in individuals with nonsyndromic hearing loss

Jinsei Jung, Hyun Been Choi, Young Ik Koh, John Hoon Rim, Hye Ji Choi, Sung Huhn Kim, Jae Hyun Lee, Jieun An, Ami Kim, Joon Suk Lee, Sun Young Joo, Seyoung Yu, Jae Young Choi, Tong Mook Kang, Heon Yung Gee

Table S1. Pathogenic or likely pathogenic *KCNQ4* variants linked to DFNA2.

Nucleotide change	Amino acid change	HG-MD	ClinVar	Reference
c.211del	p.Q71Sfs*68	O	Pathogenic	Kamada (2006) J Hum Genet 51: 455 PubMed: 16596322
c.212_224del	p.Q71Pfs*64	O		Coucke (1999) Hum Mol Genet 8: 1321 PubMed: 10369879
c.228_229dup	p.H77Rfs*63	O	Pathogenic	Naito (2013) PLoS One 8: e63231 PubMed: 23717403
c.459del	p.A154Pfs*85		Pathogenic	
c.463G>A	p.G155R	O		Iwasa (2016) PLoS One 11: e0166781 PubMed: 27911912
c.546C>G	p.F182L	O	Pathogenic	Su (2007) Audiol Neurootol 12: 20 PubMed: 17033161
c.648C>T	synonymous	O		Su (2007) Audiol Neurootol 12: 20 PubMed: 17033161
c.650T>A	p.M217K	O		Sloan-Heggen (2016) Hum Genet 135: 441 PubMed: 26969326
c.667_684del	p.T223_G228del	O		Baek (2010) Biochim Biophys Acta 1812: 536 PubMed: 20832469
c.689T>A	p.Val230Glu	O	Pathogenic	Naito (2013) PLoS One 8: e63231 PubMed: 23717403
c.701A>T	p.H234L	O		Uehara (2015) Hum Genome Var 2: 15038 PubMed: 27081546
c.701A>G	p.His234Arg		Likely pathogenic	1Hildebrand (2008) Genet Med 10: 797 PubMed: 18941426
c.725G>A	p.W242*	O		Iwasa (2016) PLoS One 11: e0166781 PubMed: 27911912
c.754G>C	p.A252P	O		Hildebrand (2008) Genet Med 10: 797 PubMed: 18941426
c.778G>A	p.Glu260Lys	O	Pathogenic	Hildebrand (2008) Genet Med 10: 797 PubMed: 18941426
c.785A>T	p.Asp262Val	O	Pathogenic	Watabe (2013) Biochem Biophys Res Commun 432: 475 PubMed: 23399560
c.806_808delCCT	p.Ser269del	O	Likely pathogenic	Namba (2012) BMC Res Notes 5: 145 PubMed: 22420747
c.808T>C	p.Tyr270His	O	Pathogenic	van Hauwe (2000) Am J Med Genet 93: 184 PubMed: 10925378
c.821T>A	p.Leu274His	O	Pathogenic	Wang (2014) PLoS One 9: e103133 PubMed: 25116015
c.823T>C	p.Trp275Arg	O	Pathogenic	Hildebrand (2008) Genet Med 10: 797 PubMed: 18941426
c.824G>C	p.Trp275Ser		Likely pathogenic	

c.827G>C	p.Trp276Ser	O	Pathogenic	Coucke (1999) Hum Mol Genet 8: 1321 PubMed: 10369879
c.827G>T	p.W276L	O		Sommen (2016) Hum Mutat 37: 812 PubMed: 27068579
c.842T>C	p.Leu281Ser	O	Pathogenic	Talebizadeh (1999) Hum Mutat 14: 493 PubMed: 10571947
c.853G>A	p.Gly285Ser	O	Likely pathogenic	Kubisch (1999) Cell 96: 437 PubMed: 10025409
c.853G>T	p.Gly285Cys	O	Pathogenic	Coucke (1999) Hum Mol Genet 8: 1321 PubMed: 10369879
c.857A>G	p.Y286C	O		Sloan-Heggen (2016) Hum Genet 135: 441 PubMed: 26969326
c.859G>C	p.G287R	O		Arnett (2011) Arch Otolaryngol Head Neck Surg 137: 54 PubMed: 21242547
c.871C>T	p.Pro291Ser	O	Pathogenic	Naito (2013) PLoS One 8: e63231 PubMed: 23717403
c.872C>T	p.Pro291Leu	O	Pathogenic	Naito (2013) PLoS One 8: e63231 PubMed: 23717403
c.886G>A	p.Gly296Ser	O	Pathogenic	Mencía (2008) Hum Genet 123: 41 PubMed: 18030493
c.887G>A	p.G296D	O		Huang (2017) BMC Med Genet 18: 36 PubMed: 28340560
c.891G>T	p.Arg297Ser	O	Pathogenic	Naito (2013) PLoS One 8: e63231 PubMed: 23717403
c.961G>A	p.Gly321Ser	O	Pathogenic	Coucke (1999) Hum Mol Genet 8: 1321 PubMed: 10369879
c.1044_1051del	p.A349Pfs*19	O	Pathogenic	Wasano (2015) Biochem Biophys Res Commun 463: 582 PubMed: 26036578
c.1667_1671dup	p.V558Tfs*3		Likely pathogenic	
c.1725del	p.I576Sfs*40	O		Johnston (2015) Am J Hum Genet 96: 913 PubMed: 26046366
c.2014G>A	p.V672M	O		Miyagawa (2013) PLoS One 8: e71381 PubMed: 23967202
c.2039C>T	p.Ser680Phe	O	Pathogenic	Wu (2013) PLoS One 8: e57369 PubMed: 23451214

Table S2. 144 monogenic genes linked to hearing loss if mutated.

Gene Symbol	MIM number	Associated phenotypes and MIM number
ESPN	606351	**Deafness**, **autosomal recessive 36**, 609006, Autosomal recessive; **Deafness**, neurosensory, without vestibular involvement, autosomal dominant
GJB3	603324	**Deafness**, **autosomal dominant 2B**, 612644, Autosomal dominant; **Deafness**, autosomal dominant, with peripheral neuropathy; **Deafness**, autosomal recessive; **Deafness**, digenic, GJB2/GJB3, 220290, Autosomal recessive, Digenic dominant; Erythrokeratodermia variabilis et progressiva 1, 133200, Autosomal recessive, Autosomal dominant
COL9A2	120260	Epiphyseal dysplasia, multiple, 2, 600204, Autosomal dominant; **Stickler syndrome, type V**, 614284, Autosomal recessive
KCNQ4	603537	**Deafness**, **autosomal dominant 2A**, 600101, Autosomal dominant
BSND	606412	**Bartter syndrome, type 4a**, 602522, Autosomal recessive; **Sensorineural Deafness** with mild renal dysfunction, 602522, Autosomal recessive
COL11A1	120280	**Fibrochondrogenesis 1**, 228520, Autosomal recessive; {Lumbar disc herniation, susceptibility to}, 603932; **Marshall syndrome**, 154780, Autosomal dominant; **Stickler syndrome, type II**, 604841, Autosomal dominant
GPSM2	609245	**Chudley-McCullough syndrome**, 604213, Autosomal recessive
KCNJ10	602208	**Enlarged vestibular aqueduct, digenic**, 600791, Autosomal recessive; **SESAME syndrome**, 612780, Autosomal recessive
USH2A	608400	Retinitis pigmentosa 39, 613809; **Usher syndrome, type 2A**, 276901, Autosomal recessive
OTOF	603681	Auditory neuropathy, autosomal recessive, 1, 601071, Autosomal recessive; **Deafness**, **autosomal recessive 9,** 601071, Autosomal recessive
PNPT1	610316	Combined oxidative phosphorylation deficiency 13, 614932, Autosomal recessive; **Deafness**, **autosomal recessive 70**, 614934, Autosomal recessive
ELMOD3	615427	**Deafness**, **autosomal recessive 88**, 615429, Autosomal recessive
DFNB59 (PJVK)	610219	**Deafness**, **autosomal recessive 59**, 610220, Autosomal recessive
PAX3	606597	Craniofacial-**Deafness**-hand syndrome, 122880, Autosomal dominant; Rhabdomyosarcoma 2, alveolar, 268220, Autosomal recessive; **Waardenburg syndrome, type 1**, 193500, Autosomal dominant; **Waardenburg syndrome, type 3**, 148820, Autosomal recessive, Autosomal dominant
COL4A4	120131	**Alport syndrome**, autosomal recessive, 203780, Autosomal recessive; Hematuria, familial benign
COL4A3	120070	**Alport syndrome**, autosomal dominant, 104200, Autosomal dominant; Alport syndrome, autosomal recessive, 203780, Autosomal recessive; Hematuria, benign familial, 141200, Autosomal dominant
LARS2	604544	?Hydrops, lactic acidosis, and sideroblastic anemia, 617021, Autosomal recessive; **Perrault syndrome 4**, 615300, Autosomal recessive
TMIE	607237	**Deafness**, **autosomal recessive 6**, 600971, Autosomal recessive
MITF	156845	COMMAD syndrome, 617306, Autosomal recessive; {Melanoma, cutaneous malignant, susceptibility to, 8}, 614456; Tietz albinism-**Deafness** syndrome, 103500, Autosomal dominant; Waardenburg syndrome, type 2A, 193510, Autosomal dominant; Waardenburg syndrome/ocular albinism, digenic, 103470, Autosomal dominant
ILDR1	609739	**Deafness**, **autosomal recessive 42**, 609646, Autosomal recessive
CLRN1	606397	Retinitis pigmentosa 61, 614180; Usher syndrome, type 3A, 276902, Autosomal recessive
CCDC50	611051	?**Deafness**, **autosomal dominant 44**, 607453, Autosomal dominant
WFS1	606201	?Cataract 41, 116400, Autosomal dominant; **Deafness**, autosomal dominant 6/14/38, 600965, Autosomal dominant;

		{Diabetes mellitus, noninsulin-dependent, association with}, 125853, Autosomal dominant; Wolfram syndrome 1, 222300, Autosomal recessive; Wolfram-like syndrome, autosomal dominant, 614296, Autosomal dominant
GRXCR1	613283	**Deafness**, **autosomal recessive 25**, 613285, Autosomal recessive
DSPP	125485	**Deafness**, **autosomal dominant 39**, with dentinogenesis, 605594, Autosomal dominant; Dentin dysplasia, type II, 125420, Autosomal dominant; Dentinogenesis imperfecta, Shields type II, 125490, Autosomal dominant; Dentinogenesis imperfecta, Shields type III, 125500, Autosomal dominant
MARVELD2	610572	**Deafness**, **autosomal recessive 49**, 610153, Autosomal recessive
ADGRV1	602851	?Febrile seizures, familial, 4, 604352, Autosomal dominant; Usher syndrome, type 2C, 605472, Autosomal recessive, Digenic dominant; Usher syndrome, type 2C, GPR98/PDZD7 digenic, 605472, Autosomal recessive, Digenic dominant
HSD17B4	601860	D-bifunctional protein deficiency, 261515, Autosomal recessive; Perrault syndrome 1, 233400, Autosomal recessive
HARS2	600783	?Perrault syndrome 2, 614926, Autosomal recessive
DIAPH1	602121	**Deafness**, **autosomal dominant 1**, 124900, Autosomal dominant; Seizures, cortical blindness, microcephaly syndrome, 616632, Autosomal recessive
GRXCR2	615762	?**Deafness, autosomal recessive 101**, 615837, Autosomal recessive
POU4F3	602460	**Deafness**, **autosomal dominant 15**, 602459, Autosomal dominant
TCOF1	606847	Treacher Collins syndrome 1, 154500, Autosomal dominant
FOXI1	601093	Enlarged vestibular aqueduct, 600791, Autosomal recessive
SERPINB6	173321	?**Deafness**, **autosomal recessive 91**, 613453, Autosomal recessive
DCDC2	605755	?**Deafness**, **autosomal recessive 66**, 610212, Autosomal recessive; Nephronophthisis 19, 616217, Autosomal recessive; Sclerosing cholangitis, neonatal, 617394, Autosomal recessive
RIPOR2	611410	?**Deafness**, **autosomal recessive 104**, 616515, Autosomal recessive
COL11A2	120290	**Deafness**, **autosomal dominant 13**, 601868, Autosomal dominant; **Deafness**, autosomal recessive 53, 609706, Autosomal recessive; Fibrochondrogenesis 2, 614524, Autosomal recessive, Autosomal dominant; Otospondylomegaepiphyseal dysplasia, autosomal dominant, 184840, Autosomal dominant; Otospondylomegaepiphyseal dysplasia, autosomal recessive, 215150, Autosomal recessive
LHFPL5	609427	**Deafness**, **autosomal recessive 67**, 610265, Autosomal recessive
POLR1C	610060	Leukodystrophy, hypomyelinating, 11, 616494, Autosomal recessive; Treacher Collins syndrome 3, 248390, Autosomal recessive
CLIC5	607293	?**Deafness**, **autosomal recessive 103**, 616042, Autosomal recessive
COL9A1	120210	?Epiphyseal dysplasia, multiple, 6, 614135, Autosomal dominant; Stickler syndrome, type IV, 614134
MYO6	600970	**Deafness**, **autosomal dominant 22**, 606346, Autosomal dominant; **Deafness**, **autosomal dominant 22**, with hypertrophic cardiomyopathy, 606346, Autosomal dominant; **Deafness**, **autosomal recessive 37**, 607821, Autosomal recessive
EYA4	603550	Cardiomyopathy, dilated, 1J, 605362; **Deafness**, autosomal dominant 10, 601316, Autosomal dominant
GSDME	608798	**Deafness**, **autosomal dominant 5**, 600994, Autosomal dominant
ADCY1	103072	?**Deafness**, **autosomal recessive 44**, 610154, Autosomal recessive
HGF	142409	**Deafness**, **autosomal recessive 39**, 608265, Autosomal recessive
SEMA3E	608166	?CHARGE syndrome, 214800, Autosomal dominant
SLC26A5	604943	?**Deafness**, **autosomal recessive 61**, 613865, Autosomal recessive
SLC26A4	605646	**Deafness**, **autosomal recessive 4**, with enlarged vestibular aqueduct, 600791, Autosomal recessive; Pendred

		syndrome, 274600, Autosomal recessive
MET	164860	?**Deafness**, **autosomal recessive 97**, 616705, Autosomal recessive; Hepatocellular carcinoma, childhood type, somatic, 114550; {Osteofibrous dysplasia, susceptibility to}, 607278, Autosomal dominant; Renal cell carcinoma, papillary, 1, familial and somatic, 605074
MIR96	611606	**Deafness**, **autosomal dominant 50**, 613074, Autosomal dominant
SNAI2	602150	Piebaldism, 172800, Autosomal dominant; Waardenburg syndrome, type 2D, 608890, Autosomal recessive
CHD7	608892	CHARGE syndrome, 214800, Autosomal dominant; Hypogonadotropic hypogonadism 5 with or without anosmia, 612370, Autosomal dominant
EYA1	601653	Anterior segment anomalies with or without cataract, 602588, Autosomal dominant; Branchiootic syndrome 1, 602588, Autosomal dominant; Branchiootorenal syndrome 1, with or without cataracts, 113650, Autosomal dominant; ?Otofaciocervical syndrome, 166780, Autosomal dominant
GRHL2	608576	**Deafness**, **autosomal dominant 28**, 608641, Autosomal dominant; Ectodermal dysplasia/short stature syndrome, 616029, Autosomal recessive
MTAP	156540	Diaphyseal medullary stenosis with malignant fibrous histiocytoma, 112250, Autosomal dominant
TJP2	607709	Cholestasis, progressive familial intrahepatic 4, 615878, Autosomal recessive; Hypercholanemia, familial, 607748
TMC1	606706	**Deafness**, **autosomal dominant 36**, 606705, Autosomal dominant; **Deafness**, autosomal recessive 7, 600974, Autosomal recessive
WHRN	607928	**Deafness**, **autosomal recessive 31**, 607084, Autosomal recessive; Usher syndrome, type 2D, 611383, Autosomal recessive
TNC	187380	**Deafness**, **autosomal dominant 56**, 615629, Autosomal dominant
TPRN	613354	**Deafness**, **autosomal recessive 79**, 613307, Autosomal recessive
MYO3A	606808	**Deafness**, **autosomal recessive 30**, 607101, Autosomal recessive
PCDH15	605514	**Deafness**, **autosomal recessive 23**, 609533, Autosomal recessive; Usher syndrome, type 1D/F digenic, 601067, Autosomal recessive, Digenic recessive; Usher syndrome, type 1F, 602083, Autosomal recessive
CDH23	605516	**Deafness**, **autosomal recessive 12**, 601386, Autosomal recessive; {Pituitary adenoma 5, multiple types}, 617540, Autosomal dominant; Usher syndrome, type 1D, 601067, Autosomal recessive, Digenic recessive; Usher syndrome, type 1D/F digenic, 601067, Autosomal recessive, Digenic recessive
PDZD7	612971	{Retinal disease in Usher syndrome type IIA, modifier of}, 276901, Autosomal recessive; Usher syndrome, type IIC, GPR98/PDZD7 digenic, 605472, Autosomal recessive, Digenic dominant
KCNQ1	607542	Atrial fibrillation, familial, 3, 607554, Autosomal dominant; Jervell and Lange-Nielsen syndrome, 220400, Autosomal recessive; Long QT syndrome 1, 192500, Autosomal dominant; {Long QT syndrome 1, acquired, susceptibility to}, 192500, Autosomal dominant; Short QT syndrome 2, 609621, Autosomal dominant
USH1C	605242	**Deafness**, **autosomal recessive 18A**, 602092, Autosomal recessive; Usher syndrome, type 1C, 276904, Autosomal recessive
OTOG	604487	**Deafness**, **autosomal recessive 18B**, 614945, Autosomal recessive
CABP2	607314	**Deafness**, **autosomal recessive 93**, 614899, Autosomal recessive
LRTOMT	612414	**Deafness**, **autosomal recessive 63**, 611451, Autosomal recessive
MYO7A	276903	**Deafness**, **autosomal dominant 11**, 601317, Autosomal dominant; **Deafness**, autosomal recessive 2, 600060, Autosomal recessive; Usher syndrome, type 1B, 276900, Autosomal recessive
NARS2	612803	Combined oxidative phosphorylation deficiency 24, 616239, Autosomal recessive

RDX	179410	**Deafness**, **autosomal recessive 24**, 611022, Autosomal recessive
TECTA	602574	**Deafness**, **autosomal dominant 8/12**, 601543, Autosomal dominant; **Deafness**, **autosomal recessive 21**, 603629, Autosomal recessive
EPS8	600206	?**Deafness**, **autosomal recessive 102**, 615974, Autosomal recessive
COL2A1	120140	Achondrogenesis, type II or hypochondrogenesis, 200610, Autosomal dominant; Avascular necrosis of the femoral head, 608805, Autosomal dominant; Czech dysplasia, 609162, Autosomal dominant; Epiphyseal dysplasia, multiple, with myopia and **Deafness**, 132450, Autosomal dominant; Kniest dysplasia, 156550, Autosomal dominant; Legg-Calve-Perthes disease, 150600, Autosomal dominant; Osteoarthritis with mild chondrodysplasia, 604864, Autosomal dominant; Platyspondylic skeletal dysplasia, Torrance type, 151210, Autosomal dominant; SED congenita, 183900, Autosomal dominant; SMED Strudwick type, 184250, Autosomal dominant; Spondyloepiphyseal dysplasia, Stanescu type, 616583, Autosomal dominant; Spondyloperipheral dysplasia, 271700, Autosomal dominant; Stickler sydrome, type I, nonsyndromic ocular, 609508, Autosomal dominant; Stickler syndrome, type I, 108300, Autosomal dominant; Vitreoretinopathy with phalangeal epiphyseal dysplasia
MYO1A	601478	Previously known as Deafness, AUTOSOMAL DOMINANT 48, but reclassified
MSRB3	613719	**Deafness**, **autosomal recessive 74**, 613718, Autosomal recessive
OTOGL	614925	**Deafness**, **autosomal recessive 84B**, 614944, Autosomal recessive
PTPRQ	603317	**Deafness**, **autosomal dominant 73**, 617663, Autosomal dominant; **Deafness**, **autosomal recessive 84A**, 613391, Autosomal recessive
SLC17A8	607557	**Deafness**, **autosomal dominant 25**, 605583, Autosomal dominant
DIABLO	605219	**Deafness**, **autosomal dominant 64**, 614152, Autosomal dominant
P2RX2	600844	**Deafness**, **autosomal dominant 41**, 608224, Autosomal dominant
GJB2	121011	Bart-Pumphrey syndrome, 149200, Autosomal dominant; **Deafness**, **autosomal dominant 3A**, 601544, Autosomal dominant; **Deafness**, **autosomal recessive 1A**, 220290, Autosomal recessive, Digenic dominant; Hystrix-like ichthyosis with **Deafness**, 602540, Autosomal dominant; Keratitis-ichthyosis-**Deafness** syndrome, 148210, Autosomal dominant; Keratoderma, palmoplantar, with **Deafness**, 148350, Autosomal dominant; Vohwinkel syndrome, 124500, Autosomal dominant
GJB6	604418	**Deafness**, **autosomal dominant 3B**, 612643, Autosomal dominant; **Deafness**, **autosomal recessive 1B**, 612645, Autosomal recessive; **Deafness**, digenic GJB2/GJB6, 220290, Autosomal recessive, Digenic dominant; Ectodermal dysplasia 2, Clouston type, 129500, Autosomal dominant
POLR1D	613715	**Treacher Collins syndrome 2**, 613717, Autosomal recessive, Autosomal dominant
DIAPH3	614567	**Auditory neuropathy**, autosomal dominant, 1, 609129, Autosomal dominant
EDNRB	131244	**ABCD syndrome**, 600501, Autosomal recessive; {Hirschsprung disease, susceptibility to, 2}, 600155, Autosomal dominant; **Waardenburg syndrome, type 4A**, 277580, Autosomal recessive, Autosomal dominant
COCH	603196	**Deafness**, **autosomal dominant 9**, 601369, Autosomal dominant
SIX1	601205	Branchiootic syndrome 3, 608389, Autosomal dominant; **Deafness**, autosomal dominant 23, 605192, Autosomal dominant
ESRRB	602167	**Deafness**, **autosomal recessive 35**, 608565, Autosomal recessive
STRC	606440	**Deafness**, **autosomal recessive 16**, 603720, Autosomal recessive
CIB2	605564	**Deafness**, **autosomal recessive 48**, 609439, Autosomal recessive; Usher syndrome, type IJ, 614869, Autosomal recessive

HOMER2	604799	?**Deafness**, **autosomal dominant 68**, 616707, Autosomal dominant
TBC1D24	613577	DOORS syndrome, 220500, Autosomal recessive; **Deafness** , **autosomal recessive 86**, 614617, Autosomal recessive; **Deafness**, **autosomal dominant 65**, 616044, Autosomal dominant; Epileptic encephalopathy, early infantile, 16, 615338, Autosomal recessive; Myoclonic epilepsy, infantile, familial, 605021, Autosomal recessive
CRYM	123740	**Deafness**, **autosomal dominant 40**, 616357, Autosomal dominant
OTOA	607038	**Deafness**, **autosomal recessive 22**, 607039, Autosomal recessive
KARS	601421	?Charcot-Marie-Tooth disease, recessive intermediate, B, 613641, Autosomal recessive; **Deafness**, **autosomal recessive 89**, 613916, Autosomal recessive
MYO15A	602666	**Deafness**, **autosomal recessive 3**, 600316, Autosomal recessive
TMEM132E	616178	Li et al. Hum Mutat. 2015;36(1):98-105
USH1G	607696	**Usher syndrome, type 1G**, 606943, Autosomal recessive
ACTG1	102560	Baraitser-Winter syndrome 2, 614583, Autosomal dominant; **Deafness**, **autosomal dominant 20/26**, 604717, Autosomal dominant
LOXHD1	613072	**Deafness**, **autosomal recessive 77**, 613079, Autosomal recessive
GIPC3	608792	**Deafness**, **autosomal recessive 15**, 601869, Autosomal recessive
CLPP	601119	Perrault syndrome 3, 614129, Autosomal recessive
S1PR2	605111	**Deafness**, **autosomal recessive 68**, 610419, Autosomal recessive
SYNE4	615535	**Deafness**, **autosomal recessive 76**, 615540, Autosomal recessive
CEACAM16	614591	**Deafness**, **autosomal dominant 4B**, 614614, Autosomal dominant
SIX5	600963	**Branchiootorenal syndrome 2**, 610896
MYH14	608568	**Deafness**, **autosomal dominant 4A**, 600652, Autosomal dominant; **Peripheral neuropathy**, **myopathy, hoarseness, and hearing loss**, 614369, Autosomal dominant
EDN3	131242	Central hypoventilation syndrome, congenital, 209880, Autosomal dominant; {Hirschsprung disease, susceptibility to, 4}, 613712, Autosomal dominant; **Waardenburg syndrome, type 4B**, 613265, Autosomal recessive, Autosomal dominant
OSBPL2	606731	**Deafness**, **autosomal dominant 67**, 616340, Autosomal dominant
KCNE1	176261	**Jervell and Lange-Nielsen syndrome 2**, 612347, Autosomal recessive; Long QT syndrome 5, 613695, Autosomal dominant
CLDN14	605608	**Deafness**, **autosomal recessive 29**, 614035, Autosomal recessive
TMPRSS3	605511	**Deafness**, **autosomal recessive 8/10**, 601072, Autosomal recessive
TSPEAR	612920	**Deafness**, **autosomal recessive 98**, 614861, Autosomal recessive
MYH9	160775	**Deafness**, **autosomal dominant 17**, 603622, Autosomal dominant; Epstein syndrome, 153650, Autosomal dominant; Fechtner syndrome, 153640, Autosomal dominant; Macrothrombocytopenia and **progressive Sensorineural Deafness**, 600208, Autosomal dominant; May-Hegglin anomaly, 155100, Autosomal dominant; Sebastian syndrome, 605249, Autosomal dominant
TRIOBP	609761	**Deafness**, **autosomal recessive 28**, 609823, Autosomal recessive
SOX10	602229	**PCWH syndrome**, 609136, Autosomal dominant; **Waardenburg syndrome, type 2E**, with or without neurologic involvement, 611584, Autosomal dominant; **Waardenburg syndrome, type 4C**, 613266, Autosomal dominant
SMPX	300226	**Deafness**, **X-linked 4**, 300066, X-linked dominant
NDP	300658	Exudative vitreoretinopathy 2, X-linked, 305390; **Norrie disease**, 310600, X-linked recessive

POU3F4	300039	**Deafness**, **X-linked 2**, 304400, X-linked recessive
PRPS1	311850	Arts syndrome, 301835, X-linked recessive; Charcot-Marie-Tooth disease, X-linked recessive, 5, 311070, X-linked recessive; **Deafness**, **X-linked 1**, 304500, X-linked; Gout, PRPS-related, 300661, X-linked recessive; Phosphoribosylpyrophosphate synthetase superactivity, 300661, X-linked recessive
COL4A6	303631	?**Deafness**, **X-linked 6**, 300914, X-linked recessive
COL4A5	303630	Alport syndrome, 301050, X-linked dominant
TMEM43	612048	Arrhythmogenic right ventricular dysplasia 5, 604400, Autosomal dominant; Emery-Dreifuss muscular dystrophy 7, AD, 614302, Autosomal dominant
MPZL2	604873	Wesdorp et al. Am J Hum Genet. 2018;103(1):74-88, Bademci et al. Hum Genet. 2018;137(6-7):479-486.
CDC14A	603504	**Deafness**, **autosomal recessive 105**, 616958, Autosomal recessive
BDP1	607012	Girotto et al., PLoS One. 2013;8(12):e80323.
SLC22A4	604190	Ben Said et al., Hum Genet. 2016;135(5):513-24.
PPIP5K2	611648	Yousaf et al., PLoS Genet. 2018;14(3):e1007297.
EPS8L2	614988	**Deafness autosomal recessive 106**, 617637, Autosomal recessive
WBP2	606962	**Deafness**, **autosomal recessive 107**, 617639, Autosomal recessive
ROR1	602336	?**Deafness**, **autosomal recessive 108**, 617654, Autosomal recessive
ESRP1	612959	Rohacek et al., Dev Cell. 2017;43(3):318-331.
NLRP3	606416	CINCA syndrome, 607115, Autosomal dominant; **Deafness**, **autosomal dominant 34**, with or without inflammation, 617772, Autosomal dominant; Familial cold-induced inflammatory syndrome 1, 120100, Autosomal dominant; Muckle-Wells syndrome, 191900, Autosomal dominant
CD164	603356	?**Deafness**, **autosomal dominant 66**, 616969, Autosomal dominant
MCM2	116945	?**Deafness**, **autosomal dominant 70**, 616968, Autosomal dominant
KITLG	184745	**Deafness**, **autosomal dominant 69**, unilateral or asymmetric, 616697, Autosomal dominant; Hyperpigmentation with or without hypopigmentation, 145250, Autosomal dominant; [Skin/hair/eye pigmentation 7, blond/brown hair], 611664
DMXL2	612186	?**Deafness**, **autosomal dominant 71**, 617605, Autosomal dominant; ?Polyendocrine-polyneuropathy syndrome, 616113, Autosomal recessive
COL9A3	120270	Epiphyseal dysplasia, multiple, 3, with or without myopathy, 600969, Autosomal dominant; {Intervertebral disc disease, susceptibility to}, 603932
HARS	142810	Charcot-Marie-Tooth disease, axonal, type 2W, 616625, Autosomal dominant; **Usher syndrome type 3B**, 614504, Autosomal recessive
ERAL1	607435	**Perrault syndrome 6**, 617565, Autosomal recessive

Table S3. Filtering process of whole-exome sequencing analysis.

Individual	YUHL35 II-6	YUHL41 III-2
Total sequence reads	98,057,930	68,133,618
Matched reads (percentage of matched reads)	95,992,778 (97.89%)	67,823,755 (99.55%)
Total number of variants detected	205,883	179,169
Variants which are not common in the gnomAD (MAF>1%) (A)	2,374	2,314
Variants filtered by 32 internal control data	1,641	1,602
Variants which are nonsynonymous or located in splice junction (B)	550	580
% B / A	23%	25%
Located within splice site	39	36
Deletion/Insertion	11	17
Stop codon gained / Stop codon lost	5	11
Missense	479	488
Variants in 144 hearing loss known genes	12	9
Located within splice site	2	2
Deletion/Insertion	3	1
Stop codon gained / Stop codon lost	0	0
Missense	7	7

Table S4. Variants detected in YUHL35 II-6 in 144 monogenic genes linked to hearing loss

YUHL	Gene symbol	hg19	Accession #	cDNA position	Amino acid substitution	Count	Cove-rage	Conservation				dbSNP150	gnomAD MAF	NBK MAF	Mutation Taster	PP2 Humvar	SIFT	Condel	CADD	OMIM	HGMD/ ClinVar	Sanger confirmation
								Mm	Gg	Xt	Dr											
YUHL 35 II-6	***KCNQ4***	chr1:41285106_G/T	NM_004700.3	c.796G>T	p.Asp266Tyr	45	87	D	E	E	D	-	ND	ND	DC	Dam	Del	Del	32	Deafness, autosomal dominant 2A (AD)	ND/ND	Confirmed
	**San+V4:V13**	chr1:103474068_C/G	NM_001854.3	c.1634G>C	p.Gly545Ala	42	89	G	G	A	L	-	0.00002031 (E.A=0.0002900)	ND	Polymorphism	Benign	Tolerated	Neutral	7.113	Marshall syndrome (AD)/ Stickler syndrome, type II (AD)	ND/ND	not performed
	DMXL2	chr15:51868351_T/A	NM_001174116.1	c.115A>T	p.Ile39Phe	29	138	I	I	I	I	rs929082641 A=0.0090/1132 (TOPMED)	ND	ND	DC	Dam	Del	Del	31	Deafness, autosomal dominant 71 (AD)	ND/ND	not confirmed
	DMXL2	chr15:51868354_C/A	NM_001174116.1	c.112G>T	p.Val38Phe	28	133	V	V	V	V	rs751988920 A=0.00003/3 (ExAC) A=0.0012/148 (TOPMED)	ND	ND	DC	Dam	Del	Del	32	Deafness, autosomal dominant 71 (AD)	ND/ND	not confirmed
	DMXL2	chr15:51868381_A/-	NM_001174116.1	c.88-3del	-	26	26					rs774907719 -=0.1974/2528 (ExAC) -=0.0188/2364 (TOPMED)	ND	ND	-	-	-	-	15.85	Deafness, autosomal dominant 71 (AD)	ND/ND	Confirmed, but not segregated in the family
	TBC1D24	chr16:2550849_C/T	NM_001199107.1	c.1570C>T	p.Arg524Trp	73	128	R	H	Y	H	rs78644690 T=0.0003/32 (ExAC) T=0.00008/1 (GO-ESP) T=0.0004/56 (TOPMED)	0.0003134 (E.A=0.003987)	T:0.0100756	DC	Benign	Tolerated	Neutral	23.4	Deafness , autosomal recessive 86 (AR)/ Deafness, autosomal dominant 65 (AD)	ND/ Reported (Likely benign)	not performed
	COL9A3	chr20:61455815_CCAGGTCCC/-	NM_001853.3	c.543_551del	p.Pro185_Gly187del	9	30					rs765392378 -=0.0005/24 (ExAC) -=0.0003/40 (TOPMED)	0.0002316 (E.A=0.0002832)	ND	-	-	-	-	22	Epiphyseal dysplasia, multiple, 3, with or without myopathy (AD)	ND/ND	not confirmed
	USH2A	chr1:216419934_A/C	NM_206933.2	c.2802T>G	p.Cys934Trp	62	134	C	C	C	C	rs201527662 C=0.0002/26 (ExAC) C=0.0008/4 (1000 Genomes) C=0.0002/30 (TOPMED)	0.0001915 (E.A=0.002441)	C:0.00377834	DC	-	-	-	26.6	Retinitis pigmentosa 39 / Usher syndrome, type 2A(AR)	Reported (CM114180)/ Reported (Conflicting interest)	not performed
	ADGRV1	chr5:89925321_G/A	NM_032119.3	c.1804G>A	p.Ala602Thr	44	83	A	A	A	A	rs201015784 A=0.0002/22 (ExAC) A=0.0004/2 (1000 Genomes) A=0.00004/5 (TOPMED)	0.0001103 (E.A= 0.001510)	A:0.00881612	DC	Dam	Tolerated	Del	25.5	Usher syndrome, type 2C (AR)/ Usher syndrome, type 2C, GPR98/PDZD7 digenic (AR)	ND/ND	not performed
	TMIE	chr3:46751074_AAG/-	NM_147196.2	c.391_393del	p.Lys131del	17	43					rs10578999 AAG=0.2640/1303 (1000 Genomes) AAG=0.2407/30224 (TOPMED)	0.006457 (E.A=0.0006393)	ND	-	-	-	-	13.71	Deafness, autosomal recessive 6 (AR)	ND/ Reported (Conflicting interest)	not performed
	CLIC5	chr6:45922867_G/A	NM_001114086.1	c.650+5C>T	-	96	164					rs755109540 A=0.00005/6 (ExAC) A=0.00002/2 (TOPMED)	0.00006155 (S.A=0.0004887)	-	-	-	-	-	4.971	Deafness, autosomal recessive 103 (AR)	ND/ND	not performed
	TRIOBP	chr22:38120176_CCT/-	NM_001039141.2	c.1617_1619del	p.Ser540del	39	125					rs36219868 -=0.3074/36647 (ExAC) -=0.2504/1254 (1000 Genomes) -=0.2788/3273 (GO-ESP) -=0.1959/24596 (TOPMED)	0.08830 (E.A=0.2308)	ND	-	-	-	-	12.65	Deafness, autosomal recessive 28 (AR)	ND/ND	not performed

Abbreviations are as follows: CADD, Combined Annotation Dependent Depletion; Condel, CONsensus DELeteriousness score of non-synonymous single nucleotide variants; Dam, damaging; DC, disease causing; Del, deleterious; E.A, East Asian; HGMD, Human Gene Mutation Database; MAF, minor allele frequency; ND, no data ; OMIM, Online Mendelian Inheritance in Man; PD, probably damaging; PP2, PolyPhen-2 prediction score Humvar; PROVEAN, Protein Variation Effect Analyzer; S.A, South Asian; SIFT, Sorting Intolerant from Tolerant; SNP, single nucleotide polymorphism; YUHL, Yonsei University Hearing Loss cohort.
dbSNP database (http://www.ncbi.nlm.nih.gov/SNP). genome Aggregation Database browser (http://gnomad.broadinstitute.org/). fNational Biobank of Korea (NBK), Centers for Disease Control and Prevention. Mutation taster (MT) (http://www.mutationtaster.org/). PolyPhen-2 (PP2) prediction score HumVar ranges from 0 to 1.0; 0 = benign, 1.0 = probably damaging (http://genetics.bwh.harvard.edu/pph2/). SIFT (http://sift.jcvi.org/). Condel (http://bbglab.irbbarcelona.org/fannsdb/) . CADD (https://cadd.gs.washington.edu/). OMIM (https://www.omim.org/). HGMD (http://www.hgmd.cf.ac.uk/ac/index.php). ClinVar (https://www.ncbi.nlm.nih.gov/clinvar/).

YUHL35, II-6 (F/40 yr)

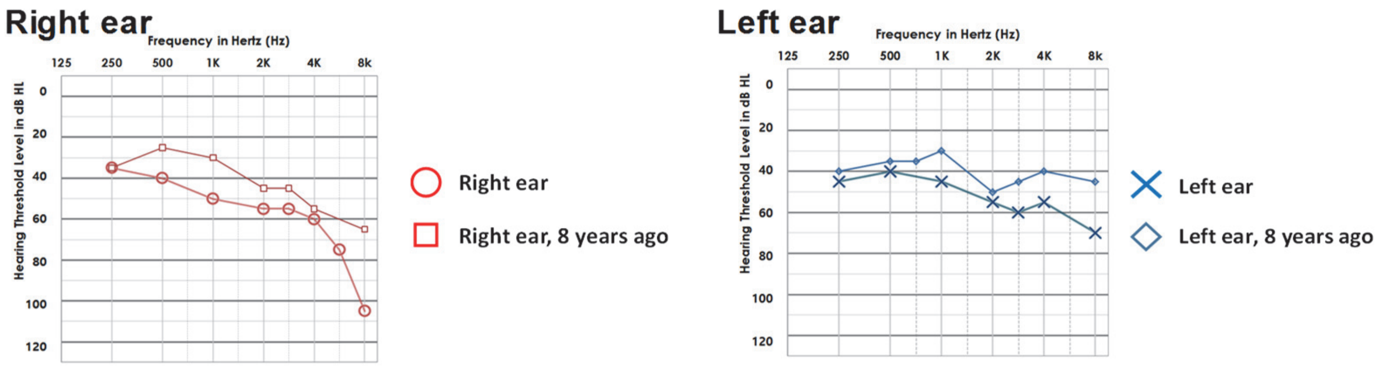

Figure S1. Long term follow-up of pure-tone audiograms in YUHL35.
Hearing thresholds in II-6 were followed-up for 8 years, after which hearing function became worse bilaterally, indicating slow progression of hearing loss.

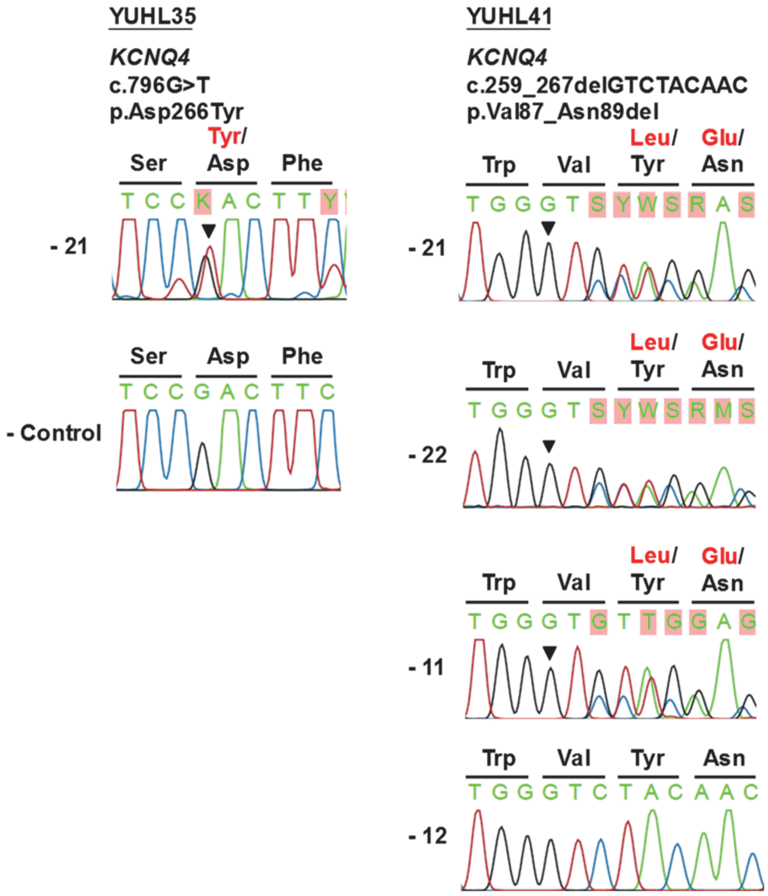

Figure S2. Sequencing traces of mutations detected in KCNQ4. Families are listed in the same order as in Table 1. Family numbers (underlined), mutated genes, altered nucleotides, and amino-acid changes are given above sequence traces. Wild-type control sequences are shown below mutated sequences. Codon triplets are underlined to indicate the reading frame. Mutated nucleotides are denoted by arrowheads.

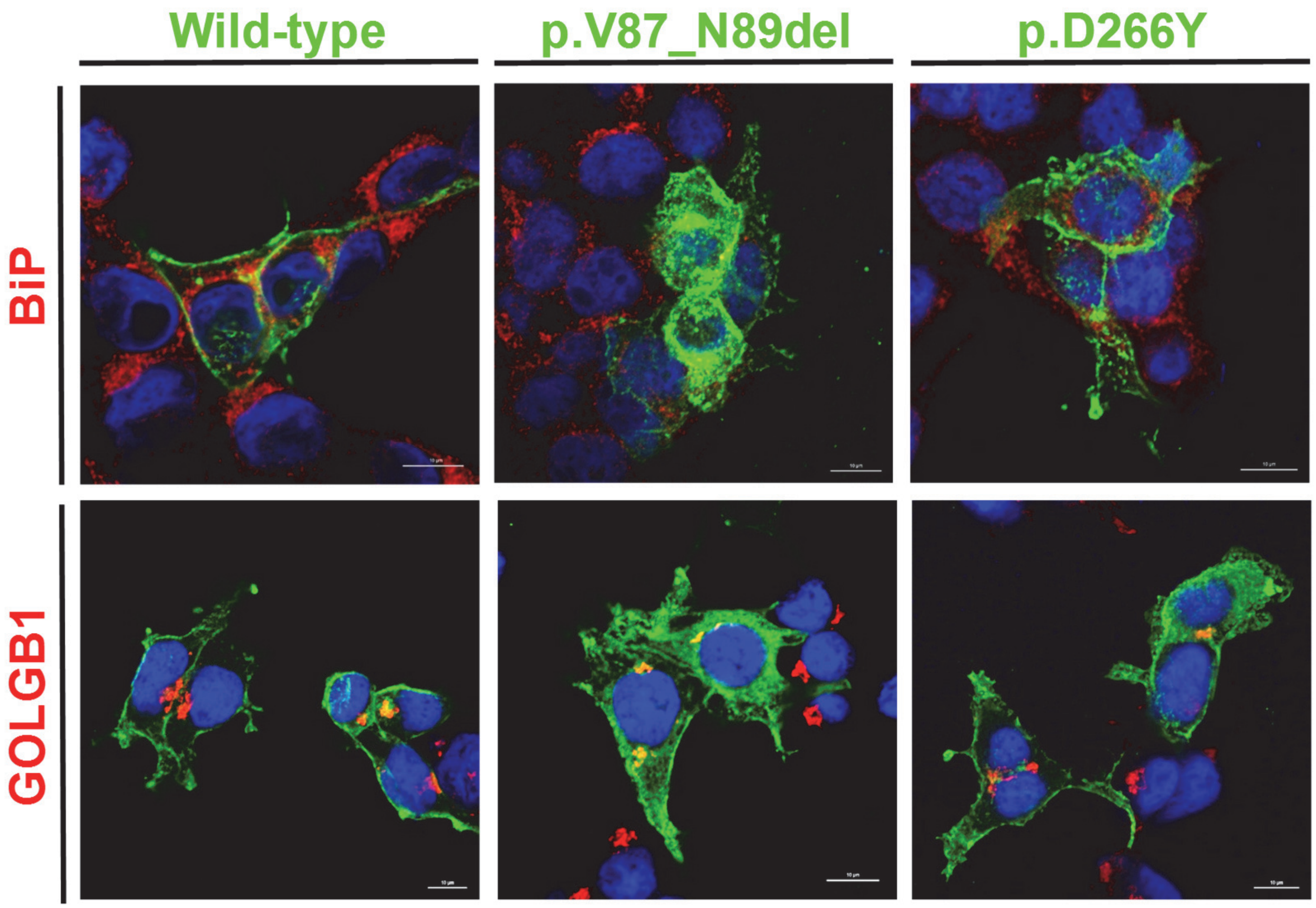

Figure S3. Immunofluorescence of wild-type and mutant KCNQ4 proteins in HEK293T cells.

HEK293T were transfected with N-terminally Myc-tagged wild-type (WT) and mutant KCNQ4 clones. Cells were immunostained with anti-Myc, anti-BiP, and anti-GOLGB1 antibodies. Nuclei were stained with DAPI. BiP and GOLGB1 are markers for the endoplasmic reticulum and Golgi apparatus, respectively. Both mutant KCNQ4 proteins and WT protein were observed on the plasma membrane.

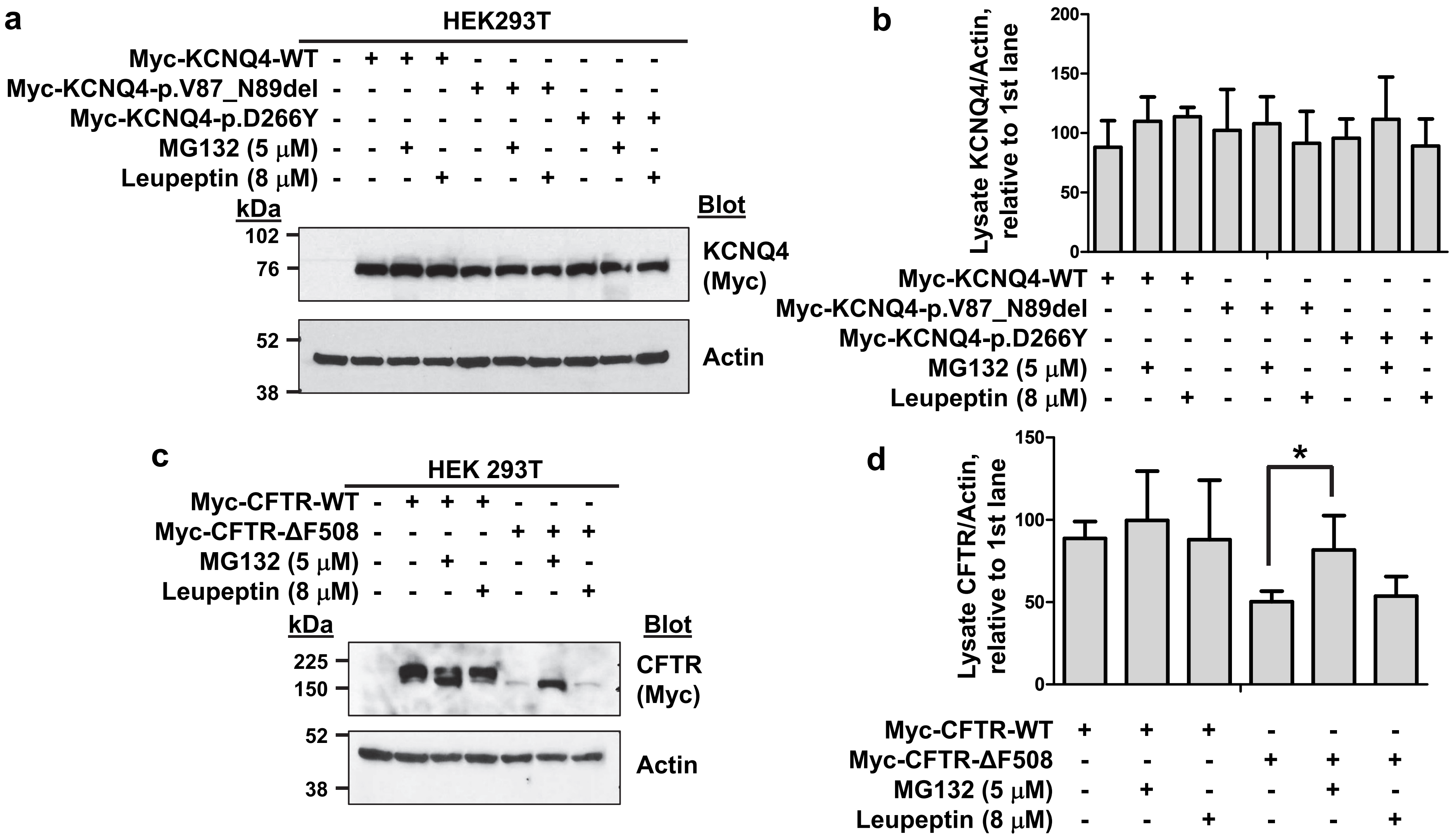

Figure S4. Effect of proteasome or lysosome inhibition on expression of KCNQ4 and CFTR. HEK293T cells were transfected with Myc-tagged KCNQ4 or CFTR wild-type (WT) mutant plasmids. Cells were treated with proteasomal inhibitor MG132 or lysosomal inhibitor leupeptin for 24 hr. (**a, b**) Effect of MG132 or leupeptin on expression of WT-, p.V87_N89del-, and p.D266Y-KCNQ4. The blot (b) is representative of three experiments and band intensity (b) was quantified as mean + SD of three experiments. Note that the expression level of either p.V98_N89del or p.D266Y KCNQ4 was not affected by MG132 or leupeptin treatment. (**c, d**) Effect of MG132 or leupeptin on expression of WT- and ΔF508-CFTR The blot (c) is representative of three experiments and band intensity (d) was quantified as mean + SD of three experiments. Note that trafficking defective mutant, ΔF508-CFTR, was significantly increased by MG132 treatment. * $P < 0.05$, t-test.

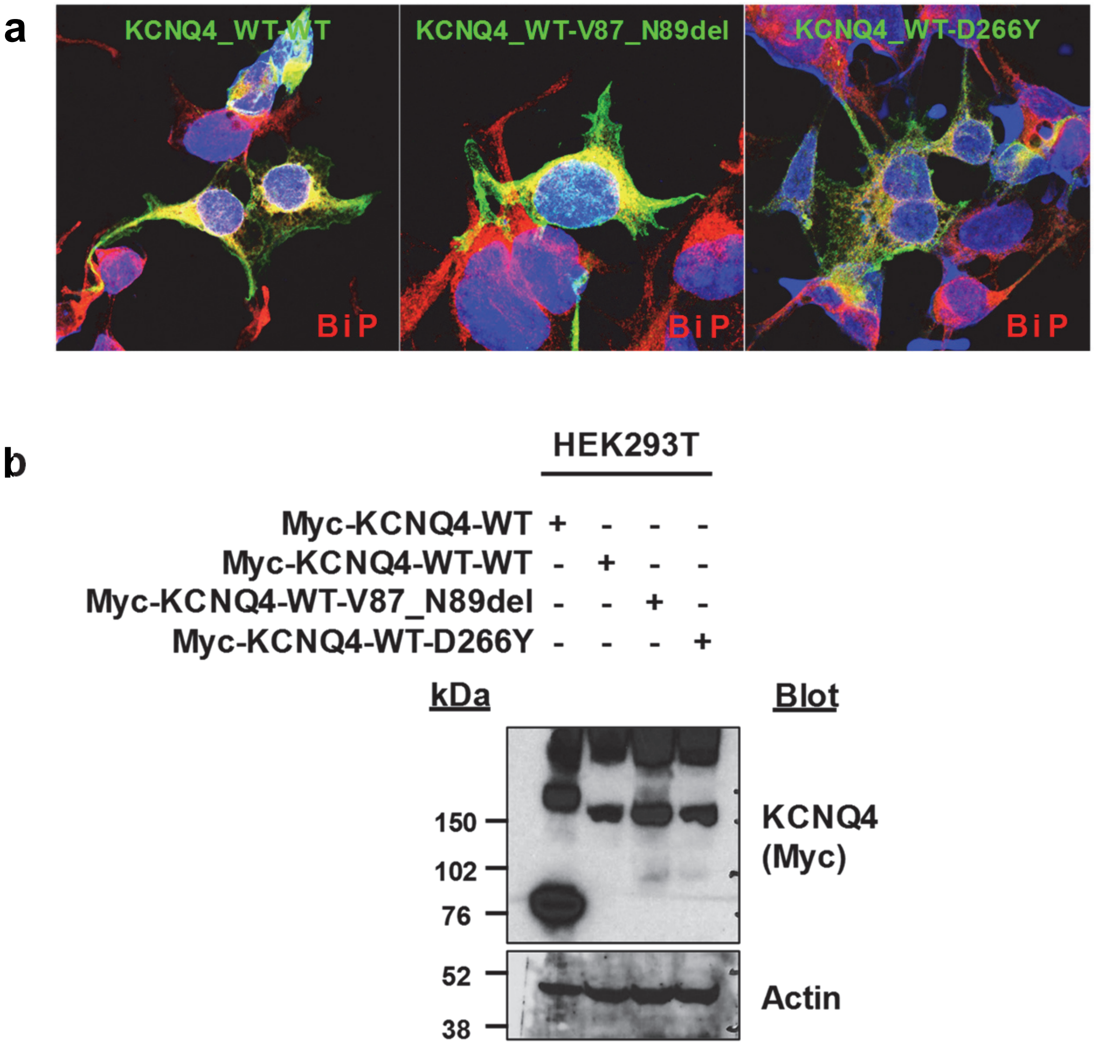

Figure S5. Expression of tandem concatamers of wild-type and mutant KCNQ4.
HEK293T cells were transfected with Myc-tagged KCNQ4 wild-type (WT)-WT and WT-mutant plasmids. (**a**) Immunofluorescence of tandem concatamers of KCNQ4 with an endoplasmic reticulum marker, BiP. (**b**) Immunoblotting of tandem concatamers. Cell lysates were blotted with anti-Myc antibodies, and 10 or 50 mg protein was loaded into each lane for KCNQ4-WT monomer and concatamers, respectively.

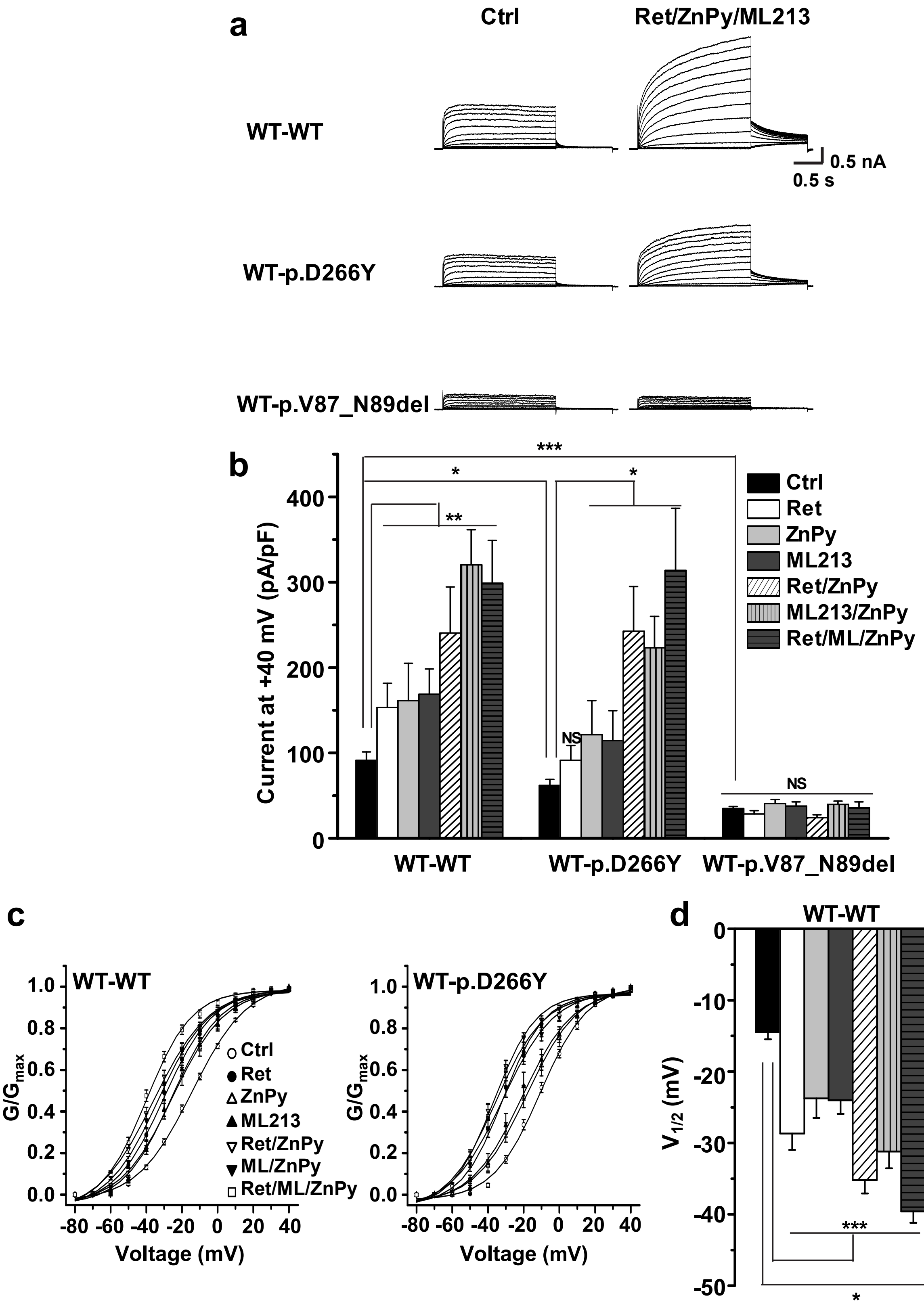

Figure S6. Effects of KCNQ openers on KCNQ4 channels assembled from tandem concatemers.
(**a**) Effects of combination treatment with KCNQ openers (10 μM Ret, 10 μM ZnPy, and 3 μM ML213) on the K^+ current from WT-WT, WT-p.D266Y, and WT-p.V87_N89del concatemers. (**b**) Bar graphs showing the degree of K^+ current increases by single or combination treatment with KCNQ openers. WT-WT, n= 65; WT-p.D266Y, n= 51; WT-p.V87_N89del, n = 46. (**c, d**) Effects of KCNQ openers on the activation curves (c) and half-activation voltages ($V_{1/2}$, d) of WT-WT and WT-p.D266Y concatemers are shown. *, $p < 0.05$; **, $p < 0.01$; ***, $p < 0.005$ versus the indicated groups.

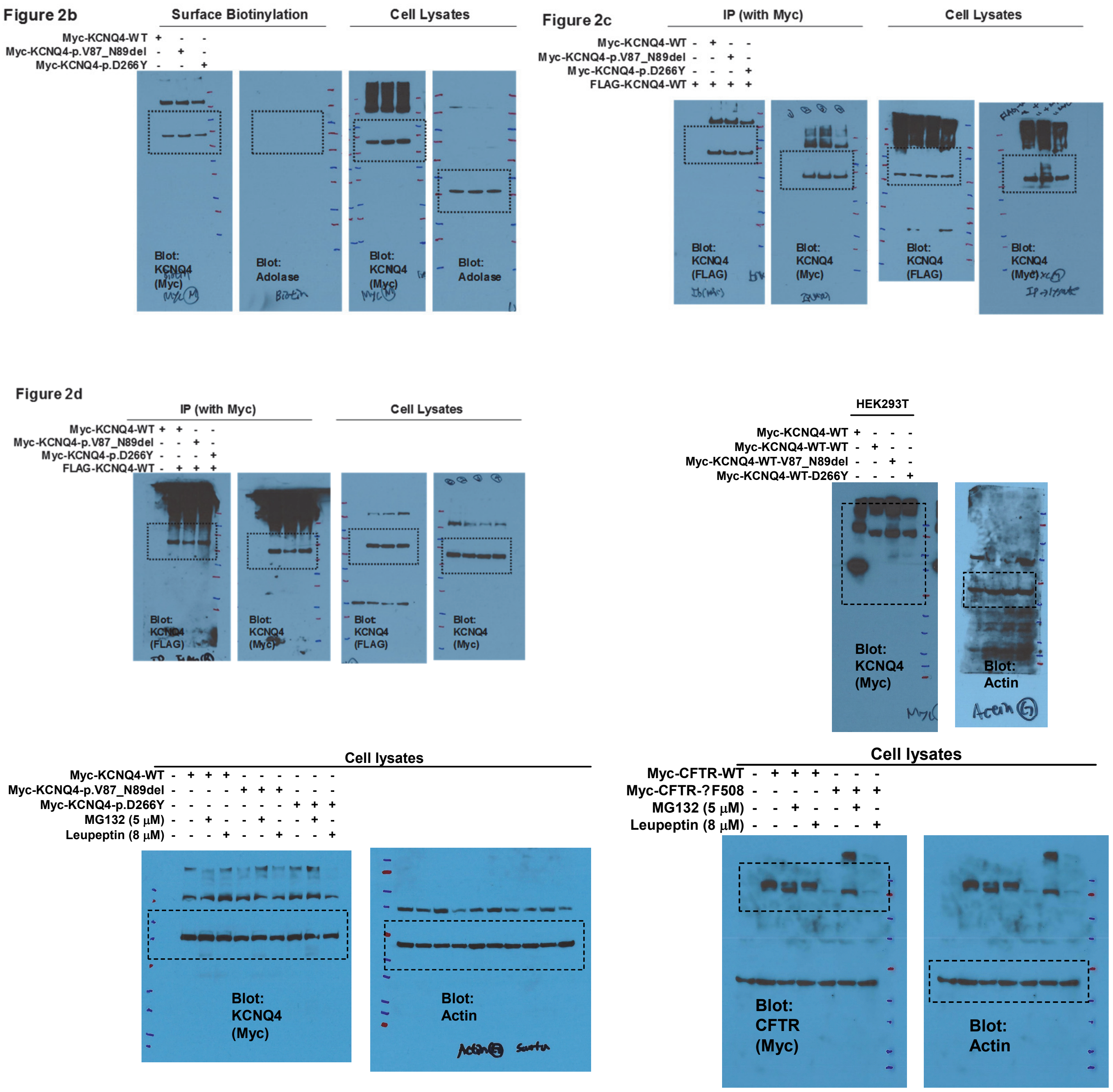

Figure S7. Uncropped images of western blot data
